# Critical Success Factors and Acceptance of the Casemix System Implementation Within the Total Hospital Information System: Exploratory Factor Analysis of a Pilot Study

**DOI:** 10.2196/56898

**Published:** 2024-10-29

**Authors:** Noor Khairiyah Mustafa, Roszita Ibrahim, Azimatun Noor Aizuddin, Syed Mohamed Aljunid, Zainudin Awang

**Affiliations:** 1 Department of Public Health Medicine Faculty of Medicine Universiti Kebangsaan Malaysia Cheras Malaysia; 2 Ministry of Health Malaysia, Precinct 1 Federal Government Administrative Centre Putrajaya Malaysia; 3 International Casemix Centre (ITCC) Hospital Canselor Tuanku Mukhriz Bandar Tun Razak Cheras Malaysia; 4 Department of Public Health and Community Medicine School of Medicine IMU University Kuala Lumpur Malaysia; 5 Faculty of Business and Management Universiti Sultan Zainal Abidin Kuala Terenggganu Malaysia

**Keywords:** critical success factors, exploratory factor analysis, Casemix system, acceptance, Total Hospital Information System

## Abstract

**Background:**

The health care landscape is evolving rapidly due to rising costs, an aging population, and the increasing prevalence of diseases. To address these challenges, the Ministry of Health of Malaysia implemented transformation strategies such as the Casemix system and hospital information system to enhance health care quality, resource allocation, and cost-effectiveness. However, successful implementation relies not just on the technology itself but on the acceptance and engagement of the users involved.

**Objective:**

This study aims to develop and refine items of a quantitative instrument measuring the critical success factors influencing acceptance of Casemix system implementation within the Ministry of Health’s Total Hospital Information System (THIS).

**Methods:**

A cross-sectional pilot study collected data from medical doctors at a hospital equipped with the THIS in the federal territory of Putrajaya, Malaysia. This pilot study’s minimum sample size was 125, achieved through proportionate stratified random sampling. Data were collected using a web-based questionnaire adapted from the human, organization, and technology-fit evaluation framework and the technology acceptance model. The pilot data were analyzed using exploratory factor analysis (EFA), and the Cronbach α assessed internal reliability. Both analyses were conducted in SPSS (version 25.0; IBM Corp).

**Results:**

This study obtained 106 valid responses, equivalent to an 84.8% (106/125) response rate. The Kaiser-Meyer-Olkin measure of sampling adequacy was 0.859, and the Bartlett test of sphericity yielded statistically significant results (*P*<.001). Principal component analysis identified 9 components explaining 84.07% of the total variance, surpassing the minimum requirement of 60%. In total, 9 unique slopes indicated the identification of 9 components through EFA. While no new components emerged from the other 7 constructs, only the organizational factors construct was divided into 2 components, later named organizational structure and organizational environment. In total, 98% (41/42) of the items had factor loadings of >0.6, leading to the removal of 1 item for the final instrument for the field study. EFA ultimately identified 8 main constructs influencing Casemix implementation within the THIS: system quality, information quality, service quality, organizational characteristics, perceived ease of use, perceived usefulness, intention to use, and acceptance. Internal reliability measured using the Cronbach α ranged from 0.914 to 0.969, demonstrating high reliability.

**Conclusions:**

This study provides insights into the complexities of EFA and the distinct dimensions underlying the constructs that influence Casemix system acceptance in the THIS. While the findings align with extensive technology acceptance literature, the results accentuate the necessity for further research to develop a consensus regarding the most critical factors for successful Casemix adoption. The developed instrument is a substantial step toward better understanding the multidimensional challenges of health care system transformations in Malaysia, postulating an underpinning for future fieldwork and broader application across other hospitals.

## Introduction

### Background

Several Ministry of Health (MOH) initiatives of the Medical Programme are proactive anticipation of further reforms to the health care system in Malaysia. This endeavor includes changing existing processes to improve hospital admission or discharge and patient flow [1-3]. These processes include the concept of clustered hospitals, lean thinking in the organization structures of hospitals, hospital information system (HIS) improvement, and using the Casemix system to measure the performance and financials [1,2]. In the case of the MOH Medical Programme, which encompasses the strategic framework for the 12th Malaysia Plan (2021-2025), this is considered one of the critical areas of concern [2]. This approach aims to enhance health system efficiency and planning with an expectation of a 13% increase in bed occupancy by 2020 [1,2].

The Medical Programme has also commenced developing and launching the Casemix system application, commonly known as the Malaysian Diagnosis-Related Group (MalaysianDRG) Casemix system. This tool sorts patients into categories by care costs, leading to improved information flow and resources [2,4,5]. It is used for cost estimation and fund allocation, and 71 hospitals have implemented it for inpatient and daycare services under the 11th Malaysia Plan, as stated in the strategic framework for the 12th Malaysia Plan [2]. Other outputs, including diagnosis-related group, severity of illness, average cost per disease, and Casemix Index can be accessed from the executive information system module [2,4,5]. The Casemix system is an imperative tool in the formulation of resource evidentiary budgets, especially for hospitals [2,4,5].

Health care finance innovations such as the Madani Medical Scheme and medical equipment rental services were designed to better the health care financing processes and application of health care services and encourage more investors to invest in the health care sector under the 12th Malaysia Plan [6]. This initiative faces a significant challenge, procuring medical equipment for major advancements in health care is costly, with high expenses related to acquisition, maintenance, and potential replacement due to ongoing innovation [6].

Hence, in order to enhance health care quality, clinical decision-making, and patient safety while reducing costs, the MOH has allocated funds for HIS and other health care information technology [7,8]. Integrating patient information facilitates the prescribing, filling, and dispensing of drugs, eliminating the need for physical prescription slips [9-11]. Studies indicate improvements in patient registration, appointment management, and ward processes, significantly reducing waiting times [12,13]. The national telehealth policy, part of the Multimedia Super Corridor initiative established 25 years ago to facilitate the country’s transformation into a high-income nation by 2020, aims to enhance patient care and expand health care information availability [3,14-16].

The HIS is a comprehensive system that integrates clinical, administrative, and financial functions to enrich service productivity [17,18]. In Malaysia, the HIS is classified into 3 types: Total HIS (THIS), Intermediate HIS, and Basic HIS [19]. Despite the importance of the HIS, only 15.2% of public hospitals in Malaysia have implemented the system within the categories of THIS, Intermediate HIS, and Basic HIS. These data indicate a low level of adoption of the HIS in the country [20].

The THIS is a comprehensive software solution that integrates patient data, administrative tasks, financial transactions, and appointment management into a single system within a hospital [1,21,22]. The THIS was developed and implemented in Selayang Hospital (1999) and Putrajaya Hospital (2000) and aimed to create a paperless digital environment [13,23]. It encompasses various applications for managing clinical notes, nursing information systems, laboratory information systems, picture archiving communication systems, radiology information systems, and pharmacy information systems [7,8,18]. The MOH of Malaysia has implemented the HIS at 37 hospitals supported by various systems and vendors (n=22). THIS hospitals use systems such as Cerner, FiSiCien, iSOFT, and ProfDoc. ProfDoc is exclusively used in a single hospital, meanwhile, Sistem Pengurusan Pesakit and SPPD are used in 9 and 10 hospitals, respectively [21]. The remaining 105 MOH hospitals operate using manual methods [21].

In addition to that, HIS@KKM, developed by the Medical Development Division, manages patient information comprehensively as part of the National Electronic Medical Record project [24]. This initiative aims to provide efficient, transparent, and prompt delivery of government health services, establishing the lifetime health record for continuous health care [24]. These initiatives are implemented to improve health care delivery by boosting productivity and efficiency, involving patients in the decision-making process, and reducing errors. Hence, strategies such as HIS and Casemix System have been introduced to support these goals [20,25,26]. Casemix has been implemented in all 149 MOH hospitals, with 19 equipped with THIS facilities since 2022 [4,5,24,27,28]. Therefore, the Casemix system within THIS facilities is either fully or partially integrated with the HIS depending on whether the vendor-supported HIS can support full integration.

**Figure 1:**
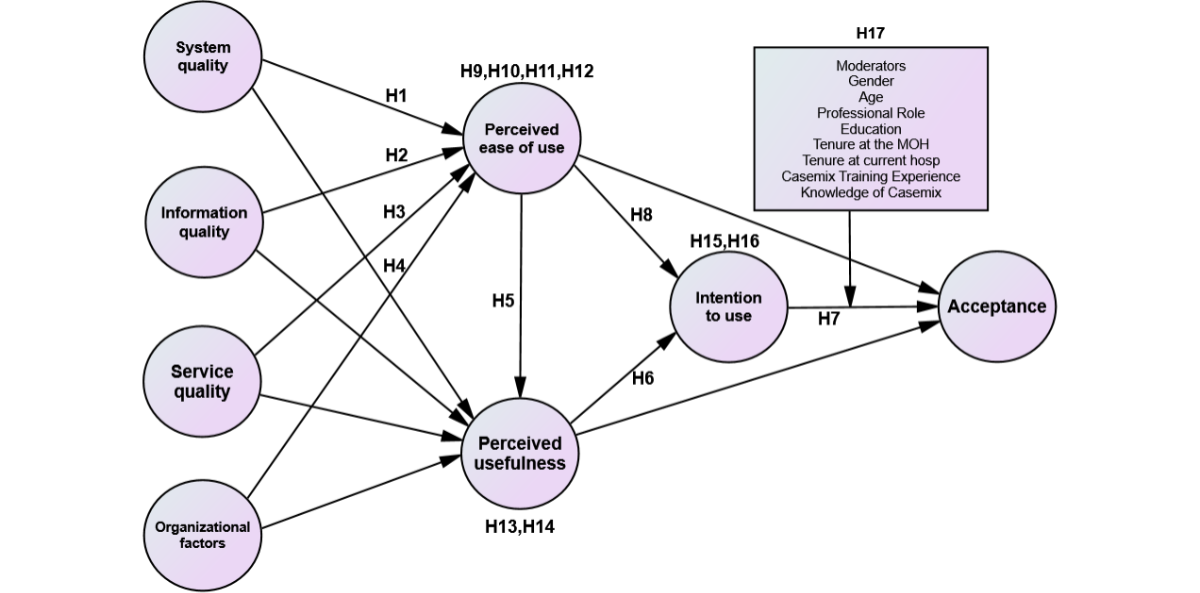
The conceptual framework. (H1-H17: Hypothesis 1 to Hypothesis 17; MOH: Ministry of Health).

Therefore, adopting and accepting new technologies in health care presents challenges due to patient vulnerability and data confidentiality concerns [29]. Various studies have identified factors affecting IT acceptability, leading to the adaptation of frameworks such as the technology acceptance model (TAM) and the human, organization, and technology-fit (HOT-Fit) evaluation framework to address these issues effectively [30-36]. Therefore, we decided to use the HOT-Fit and TAM frameworks as the conceptual basis for this study to meet its specific goals, scope, and context (Figure 1). The HOT-Fit framework provides a detailed approach to examining how human, organizational, and technological factors align, whereas the TAM focuses specifically on how individuals accept technology [31,33,37-39]. In this study, the HOT-Fit evaluation framework emphasizes technological elements such as system, information, service quality (SQ), and organizational factors, whereas the TAM addresses human aspects such as perceived ease of use (PEOU), perceived usefulness (PU), intention to use (ITU), and acceptance. Combining these frameworks is essential for meeting both specific and broad study objectives, which makes them appropriate for this research. Conversely, other models such as the Unified Theory of Acceptance and Use of Technology were deemed unsuitable due to their broad scope and complexity, and the information system success model by Delone and McLean [40] was not chosen because it is too simplistic [32,41,42].

Several studies have explored individual elements, such as system quality (SY), information quality (IQ), SQ, organizational factors, PEOU, PU, and ITU in health care information systems. However, no such studies have been known to be conducted on the Casemix system implementation within the THIS environment in Malaysia or even worldwide. A previous study was conducted on the knowledge, attitude, and practice of Casemix implementation in Turkish health care settings [43,44]. Nevertheless, many studies have been conducted in Malaysia on the HIS or even on the THIS [33-35,45-50]. Similar studies on the HIS have also been observed generally in other countries. However, no such study solely examines the THIS, which might be because the terms used in other countries differ from the HIS categories in Malaysia [10,51-56]. However, there is no specific study on Casemix integration into the THIS; hence, this constitutes the novelty of this study and the research gap [57]. Therefore, it is important to recognize that due to cultural, organizational, and structural differences in hospitals under the MOH of Malaysia, findings from other regions may not be directly applicable. Thus, a context-specific study of Casemix within the THIS is necessary to tailor the understanding to the Malaysian setting. This study involves exploring the interrelationships among these factors and their relative importance in influencing acceptance. Identifying which factors have the most significant impact and how they interact can provide deeper insights for effective implementation [57].

The critical factors in this study were predominantly adapted from the TAM and the HOT-Fit frameworks [30-36]. In addition, incorporating these elements is essential to understand their interplay and relative importance in influencing acceptance. Previous studies may have used models that partially address these factors in relation to the HIS. However, there is a need for a model that incorporates all dimensions comprehensively. Therefore, this study seeks to integrate all critical determinants and constructs into a single model based on 2 frameworks [57].

Many studies concentrate on the initial stages of system implementation, highlighting the need for longitudinal research to examine how these factors influence acceptance over time. This involves considering changes in perceptions and attitudes as users become more familiar with the system. Current researchers combine quantitative measures (eg, surveys) with qualitative insights (eg, interviews) to understand the perspectives, barriers, and challenges faced by hospital administrators and Casemix coordinators. This mixed methods approach can significantly enhance the understanding and implementation of the Casemix system in hospitals of the MOH of Malaysia, providing a model adaptable to similar contexts in other regions [57].

Exploring the critical success factors (CSFs) and acceptance of the Casemix system within Malaysia’s THIS is essential for several reasons, hence the significance of this study. First, it is imperative to stress that the presence of such factors is vital in optimizing health care processes. A smooth integration improves business continuity; therefore, practice relocation makes it possible for health care providers to spend more time treating patients than on paperwork. Gathering comprehensive patient information through an efficient and effective Casemix system is essential to ensure accurate diagnoses, effective treatment, and better patient outcomes [58]. Secondly, improved resource utilization is another advantage, ensuring that all available resources are efficiently managed. The Casemix system can help properly allocate resources as the MOH can use its financial resources more strategically [26,59,60]. This is essential when planning for the future budget and managing the costs involved. In addition, encouraging user adoption decreases the probability of apprehensions and increases satisfaction among health care employees, optimizing the use of people [48].

Third, informed decision-making is greatly improved through accurate and comprehensive data from the Casemix system. Health care administrators and policy makers can make more informed decisions regarding health care services, policies, and strategies. Continuous monitoring and enhancement of data quality leads to better clinical outcomes and patient safety, ensuring a higher standard of care [58]. In addition, improved patient outcomes result from accurate data that enable more personalized and effective patient care plans. This leads to better health outcomes and reduces the likelihood of medical errors and misdiagnoses. An efficient health information system enhances patient satisfaction by providing timely and effective health care services [48].

On top of that, this study also aids the government, especially the MOH in developing health policies and supports academic and clinical research. Insights gained from examining CSFs and acceptance can inform policies that promote the effective use of health information systems. Comprehensive data support research efforts, contributing to medical advancements and evidence-based practices [48]. On the other hand, stakeholder satisfaction, encompassing health care providers and patients, is significantly enhanced. Ensuring that the system meets the needs of health care providers increases job satisfaction and reduces turnover. For patients, the benefits of timely and effective health care services lead to higher satisfaction levels [48,58].

Furthermore, comprehending CSFs can guide future health care information technology system (HITS) implementation in other hospitals or regions. An effective implementation model can also be fine-tuned to different health care contexts and, thus, maximize positive outcomes [58]. Moreover, this study area also promotes acceptance of technological change. By calling attention to such areas, the potential for improvement and innovation in one’s own country can be achieved within HIS environment. Evidence of the ability of the Casemix system to be integrated into the health care system if the other HITS can be incorporated in the same way goes further to support the argument for developing a more synchronous HITS [48].

Finally, Casemix also has a vital role in the issue of reimbursement and financial distribution. Due to the nature of medical procedures and interventions, it also affords accurate monitoring of delivered health care services in a bid to enhance billing and reimbursement. This creates an intellectual framework that supports proper hospital reimbursement, thereby enhancing financial viability [58]. These factors collectively advance the health care system in Malaysia [48,58]. In addition, Casemix system aids in the early detection of cheaper treatment approaches and the use of available resources for a better and more economical distribution of the available financial resources [59-61]. Cost data of health care services and their demonstrable impact aid in effective financial planning to manage the hike in expenditure for health systems while maintaining or even enhancing the quality of services [59-61].

Hence, identifying CSFs and cultivating acceptance from the user group is crucial for implementing the Casemix system in the context of the health care system in the MOH of Malaysia. This way, it maintains the positive results achieved by the system, fosters better health care, manages the resources more adequately, empowers decision makers with valuable data, strengthens the positive changes in patients’ statuses, increases the satisfaction of the involved stakeholders, and positively influences financial reimbursement and allocation. These factors collectively advance the health care system in Malaysia [48,58].

Thus, the main goal of this study was to assess the CSFs and acceptance of the Casemix system implementation in the THIS of the MOH, Malaysia. Therefore, this study aimed to evaluate the appropriateness of the items using underlying constructs and investigate the reliability and validity of the study instrument (questionnaire), adapted from the HOT-Fit and TAM frameworks, in preparation for the next steps of the research process [31-35,37]. Specifically, this study had two objectives:

1. To evaluate the appropriateness of the items through the underlying factor structure in the developed quantitative instrument
2. To determine the reliability and validity of the developed quantitative instrument

By the end of this paper, the ultimate goal is to develop a valid and reliable instrument to assess the critical factors influencing the acceptance of successful Casemix system implementation within the THIS environment, in preparation for a field study.

### Theoretical and Conceptual Frameworks

The theories of the HOT-Fit highlight the significance of implementing the HIS [31,37]. According to this framework, the HIS implementation consists of 3 primary influencing dimensions: human, organization, and technology [31,37]. These components were analyzed in relation to the net benefits of the HIS implementation. The HOT-Fit evaluation framework was established and developed by Yusof et al [33,34] comprises 8 interrelated elements that determine the effectiveness of an HIS: SY, IQ, SQ, system use, user satisfaction, organizational structure, organizational environment, and net benefits. Furthermore, the TAM framework was established and developed by Davis and Venkatesh [30-32]. The TAM is supported by both theoretical and empirical evidence, suggesting that the PU and PEOU factors mitigate the impact of external variables on the ITU, assisting organizations in encouraging user adoption and use of new technologies [30-32,37].

Consequently, we selected 8 significant subdimensions from the work by Yusof et al [33-35] and Davis and Venkatesh [30,31] to create a tool gauging the CSFs and acceptance of the Casemix implementation in the THIS [30-34]. The 8 constructs include SY, IQ, SQ, organizational factors, PU, PEOU, ITU, and acceptance. The conceptual framework for this study is shown in Figure 1.

## Methods

### Study Design, Study Instrument, and Initial Validity Procedures

#### Study Design

This study adopted a sequential explanatory research design, beginning with a quantitative phase followed by a qualitative phase. However, this paper focuses on the findings from the exploratory factor analysis (EFA) conducted using data from the quantitative pilot study. The aim was to explore and refine the variables and items that measure CSFs and acceptance of the Casemix system implementation within the THIS setting, ultimately leading to the development of a robust quantitative instrument for a subsequent field study. The quantitative pilot study, conducted from February 1 to 14, 2023, used a cross-sectional approach to establish relationships between variables and evaluate underlying theories or hypotheses [62-64]. For this paper, the pilot study served as a critical step in redeveloping the quantitative instrument used, specifically within the context of a selected THIS hospital. The cross-sectional design facilitated data collection over a defined period, providing valuable insights that will inform the broader research objectives [62-64].

#### Study Instrument

This pilot study used a self-administered questionnaire (SAQ) comprising 60 items divided into 3 sections. Section 1a collected demographic information from participants, including age, gender, hospital name, professional role, educational level, work experience, and Casemix training, with 8 items in total. Section 1b assessed participants’ understanding of the Casemix system through 10 items rated on a 10-point scale, from “no knowledge at all” (1) to “exceptional/excellent knowledge” (10). These demographic and knowledge factors will later serve as moderating variables in the field study to evaluate their influence on participants’ acceptance or rejection of the Casemix implementation in the THIS environment.

Section 2 consists of 37 items representing perceived CSFs for Casemix implementation grouped into 7 constructs: SY, IQ, SQ, organizational factors, PEOU, PU, and ITU. Section 3 focuses on the dependent variable, acceptance, measured using 5 items. Both sections use a 10-point Likert scale, where 1 indicates “strongly disagree” and 10 indicates “strongly agree.” The instrument’s components were adapted from established frameworks such as the HOT-Fit evaluation framework and the TAM, ensuring comprehensive coverage of the 8 constructs (SY, IQ, SQ, organizational factors, PEOU, PU, ITU, and acceptance) [31,37,41,56,58,60,65]. These constructs underwent validation, reliability testing, and EFA as part of the study [66-69]. The operational definitions of these variables are detailed in Multimedia Appendix 1.

#### Independent Variables

SY refers to the performance attributes of a system, ensuring that it operates efficiently and reliably to meet user satisfaction. Key factors include usability, reliability, performance, security, and maintenance readiness [33,34,40,62,67,68,70-73].

In the health care information technology, IQ pertains to the quality of the data generated, processed, and shared by systems such as the Casemix system and HIS. Important aspects include correctness, completeness, consistency, timeliness, relevance, accessibility, and understandability [33,34,40,67,71,73].

SQ concerns the support provided to ensure the effective operation and use of systems such as the Casemix system and the HIS. Critical factors are responsiveness, dependability, assurance, empathy, and tangibles [33,34,67,73-75].

Organizational factors include the structure and environment of an organization, impacting the implementation and use of health care or HISs. The organizational structure covers power dynamics, communication, and task distribution, whereas the organizational environment encompasses regulatory, technological, and cultural factors [33,34,63,64,73,76].

PEOU measures how effortless users believe it is to use a system. Key components are ease of use, clear instructions, and simple interaction. It is a core concept in the TAM [30,31,37,77-79].

PU reflects the extent to which users believe that a system enhances their job performance. Elements include performance improvement, productivity, job effectiveness, and task efficiency. PU is also a key aspect of the TAM [30,31,37,77-79].

ITU indicates a user’s willingness and plans to use a system in their work. ITU is influenced by perceived benefits, ease of use, and other factors, impacting overall technology acceptance [30,31,37,77-79].

#### Dependent Variable

Acceptance represents health care professionals’ readiness and willingness to use technology in their daily work. It encompasses comfort and enthusiasm for using technology to enhance work efficiency and patient care. Understanding acceptance is crucial for effective HITS implementation and overall functionality [30,32,77,80,81].

#### Initial Validity Procedures

##### Overview

The questionnaire items were assessed for reliability and validity, with experts in the field consulted. Reliability refers to the unaffectedness of random errors, whereas validity is the accuracy of a score in representing a concept. Empirical research involves systematically examining conceptual abstractions through measurable responses to identify and explain phenomena. Validity evaluations included content, criterion, and face validity. A preliminary evaluation was conducted before the pilot test [72,74,76,82-86].

##### Content Validity

MOH professionals specializing in hospital financing in Malaysia conducted the content validity assessment. Content validity is crucial in developing new empirical measuring devices as it links abstract concepts with observable and measurable indicators. Carmines and Zeller [87] identified 2 steps: identifying the entire content domain related to the phenomena of interest and developing instrument items associated with the identified domain. The evidence of content validity can be measured using the content validity index (CVI) [83-85]. Multimedia Appendix 2 presents the suggested number of experts for content validation and its impact on the acceptable cutoff score on the CVI. The optimal method for quantifying the content validity of an assessment instrument using the CVI based on available evidence is to have a minimum of 6 experts review an instrument [88-91]. However, at least 2 expert panels are typically deemed appropriate [83]. The content validity assessment for this study included 2 experts from hospital financing (Casemix subunit) at the MOH of Malaysia. The 2 types of CVIs are named as item-CVI and scales-CVI, respectively [83-86,92]. The definitions and formulas for the CVI indexes are summarized in Multimedia Appendix 2. The relevance ratings were recoded as 1 (for scores of 3 or 4) and 0 (for scores of 1 or 2) before calculating the CVI. Multimedia Appendix 2 shows 2 experts’ item-scale relevance evaluations to demonstrate CVI calculation. Both experts validated the questionnaire contents, assigning ratings of 3 and 4, resulting in an S-CVI/Ave and S-SCVI/UA score of 1.00. In summary, a methodological approach to content validation should be undertaken based on the available data and industry best practices as it is essential to certifying the overall validity of an evaluation.

##### Criterion Validity

Criterion validity refers to the degree of correlation between a measure and other established measures for the same construct. An expert, a professor specializing in statistics and questionnaire development, evaluated the instrument’s items. This can be reviewed in Multimedia Appendix 3. Once content and criterion validity were established, the instrument underwent a meticulous back-to-back translation process from English to Malay by a highly skilled and qualified translator.

##### Face Validity

Face validity assessment was undertaken to evaluate the questionnaire’s consistency of responses, clarity, comprehensibility, ambiguity, and overall comments. The researchers acknowledged and resolved concerns before commencing the pilot study and fieldwork [72,74,91]. Following the validation process, 11 respondents were purposefully selected for face validity, also known as pretesting, to accomplish the prerequisite for face validation. These respondents must meet inclusion criteria such as those stipulated for participants in the field study. Subsequently, these respondents were excluded from participation in the quantitative field study. Before conducting the pilot study and fieldwork, the researchers considered the concerns that had been raised [93]. The face validity results can be observed in Multimedia Appendix 4.

### Study Location and Study Population

The study population of “Hospital W” consisted of 775 medical doctors by profession, encompassing hospital directors, deputy directors (medical division), consultants or specialists, medical officers, and house officers. The pilot study population possessed characteristics similar to the participants or samples involved in the subsequent quantitative field study. However, these respondents were excluded from participation in the quantitative field study. For this quantitative pilot study, the investigators purposely selected 2 major clinical departments, general surgery, and obstetrics and gynecology, to avoid the same respondents participating in the field study. These medical doctors should fulfill the inclusion criteria, similar to those of the final field study, as shown in Multimedia Appendix 5.

### Sampling Method

The pilot study was conducted using proportionate stratified random sampling, a probability sampling method that divides the total population into homogeneous groups [22,43,94,95]. The target population was selected from a hospital in Malaysia’s central or west regions, a federal territory hospital equipped with THIS facilities since 2000 and that has implemented the Casemix system since 2020. To ensure valid results, a minimum of 100 participants was used for the EFA [93,96]. The initial sample size, including 125 medical doctors from 2 major clinical departments, was chosen using proportionate stratified random sampling [97]. The final field study did not include these respondents.

Some studies suggest that 10% of the projected sample size would be enough for a pilot study, with a minimum of 10 to 30 people [98,99]. The pilot study was analyzed using the EFA method, and the researchers adopted a minimum of 100 responses for this pilot test. The researchers omitted the samples or responses from the pilot test research in the field study and conducted the research without participant or public involvement in the design, conduct, reporting, or dissemination strategies.

### Data Collection Methods

This voluntary SAQ for the quantitative pilot study was conducted on the web via Google Forms, with data automatically collected in Google Sheets to avoid issues such as low response rates and manual transcription associated with paper surveys [100,101]. The developed questionnaire can be viewed in Multimedia Appendix 6. The target population was provided access to the survey via a QR code or link through department coordinators and heads, ensuring that the survey was closed to a specific sample.

### Data Analysis

#### Data Analysis Tool

The pilot data were analyzed using the EFA method in SPSS (version 25.0; IBM Corp) [102].

#### Demographic Statistics and Knowledge Level

Descriptive statistics were analyzed using the same software. Demographic statistics and knowledge assessment of the Casemix are delineated in sections 1a and 1b, respectively, of the SAQ. Demographic data encompass pertinent variables such as age, gender, vocational roles, highest educational attainment, MOH of Malaysia and current hospital tenure, and Casemix training experience.

#### Kaiser-Meyer-Olkin Measure and Bartlett Test of Sphericity

The Kaiser-Meyer-Olkin (KMO) measure of sampling adequacy and Bartlett test of sphericity (BTOS) were conducted before the EFA was performed. The KMO test assesses the presence of multi-collinearity among items, whereas the Bartlett test detects the correlation among items [103-107]. The usefulness of the Bartlett sphericity test for factor analysis depends on the significance value, with a significance value approaching 0.000 (which is reported as *P*<.001) indicating acceptability [105-109].

#### EFA Conduct

EFA was used to analyze pilot study data, providing better results when multiple variables represent each component, whether exogenous or endogenous [93,96,105,106,109-112]. EFA allows researchers to uncover key aspects of developing theories or models from a broad set of hidden concepts rather than starting with predefined assumptions about the variables [93,96,105,106,109-112].

#### Dimensions and Total Variance

The total variance explained (TVE) is a crucial tool in evaluating the structure of a dataset [105,106,108,109,113-115]. TVE should meet or exceed the minimum requirement of 60%, thus making it an acceptable result [93,105,106,109,113,116,117].

#### Principal Component Analysis

EFA is used to identify and measure the dimensions of items assessing a construct, which can vary when transferred from different domains to a new research topic due to cultural and socioeconomic differences over time [118,119]. This study aimed to offer fresh insights by exploring a new setting and using principal component analysis (PCA) to reduce data. PCA is often part of EFA to reduce data but does not separate common and unique variations well [118,119]. In this study, PCA with varimax rotation was used to analyze data from diverse medical professionals [120]. Criteria for PCA include factors with eigenvalues of >1, factor loadings of >0.60 for practical relevance, and the absence of any item cross-loadings of >0.50. Items with loadings of >0.60 were kept as they came from an established set [93,96,111]. The scree plot was used to determine the optimal number of constructs to keep [93,96,111].

EFA differs from confirmatory factor analysis, which evaluates the effectiveness of items and validates them [69,81,82,85,92]. This study adapted existing frameworks (HOT-Fit and TAM) to a new context, focusing on Casemix system implementation within the THIS setting. EFA elucidates and condenses data by combining associated and interconnected factors, making it suitable for identifying 8 constructs: SY, IQ, SQ, organizational factors, PEOU, PU, ITU, and acceptance, in this study [31-35,37].

#### The Instrument’s Internal Reliability

Assessing the internal reliability of a study instrument ensures that it consistently measures what it is intended to measure. One common method is Cronbach α, which evaluates the internal consistency of a set of items or scales. It measures how closely related the items are as a group, indicating whether they reliably measure the same construct [121]. Reliability levels close to 1.00 indicate that the components under study can be measured accurately [122]. A Cronbach α value between 0.70 and 0.99 is generally considered adequate for reliability [122]. Some scholars suggest a Cronbach α range of 0.80 to 0.90 [123]. In social sciences, a Cronbach α value of 0.60 is widely accepted among researchers [108,124-126].

### Ethical Considerations

Ethical considerations were taken into account in this study, including ethics approval from the Medical Research Ethics Committee of the Faculty of Medicine, Universiti Kebangsaan Malaysia (JEP-2022-777); the Medical Research and Ethics Committee of the MOH of Malaysia (NMRR ID-22-02621-DKX); the Hospital Financing Unit (Casemix subunit) of the MOH of Malaysia; and the directors of the participating hospitals. The data collection process included a participant information sheet and consent form, ensuring anonymity and confidentiality according to the Helsinki Declaration. The participant information sheet form and the consent form can be found in Multimedia Appendices 7 and 8, respectively. Participants had 2 weeks to complete the survey, which took 10 to 15 minutes, with their data secured in the investigators’ password-protected systems. No incentives were offered for participation.

## Results

### Principal Analysis Results

#### Response Rate

The developed questionnaire was distributed to 125 respondents using proportionate stratified random sampling as part of the pilot study. The quantitative pilot study received 106 responses, achieving an 84.8% (106/125) response rate. This number exceeds the minimum sample size of 100 recommended by a few scholars, making the study suitable for analysis [93,105,106,109,116]. After thorough data cleaning and screening, all 106 responses were confirmed to be valid and free of missing data.

#### Demographic Statistics and Knowledge Level

This study analyzed demographic statistics and data for moderating factors, as shown in Table 1. The mean age of the respondents was 35.47 (SD 5.52) years, with 52.8% (56/106) falling within the age group of 31 to 40 years. Female participants comprised 65.1% (69/106) of the respondents. Medical officers had the highest response rate at 69.8% (74/106), and most (74/106, 69.8%) held a bachelor’s degree. A total of 71.7% (76/106) had >5 years of experience working at the MOH of Malaysia, with a mean of 9.87 (SD 5.60) years. The mean work experience at their current hospital was 6.25 (SD 4.55) years, with 49.1% (52/106) having >5 years of experience. In total, 79.2% (84/106) had attended Casemix system training. Section 1b of the questionnaire included 10 items to evaluate the comprehension level of the Casemix system on a 10-point interval scale. Most participants (71/106, 67%) demonstrated a high level of knowledge, followed by moderate and low levels. The mean knowledge level score of the Casemix system was 76.56% (SD 17.44%).

**Table 1:** Demographic profile and knowledge level of the Casemix system of the respondents (N=106).

Characteristics		Respondents, n (%)
**Age group (y; mean 35.47, SD 5.52 y)**		
	21-30	27 (25.5)
	31-40	56 (52.8)
	41-50	23 (21.7)
	≥51	0 (0)
**Gender**		
	Men	37 (34.9)
	Women	69 (65.1)
**Occupation**		
	Deputy hospital director	1 (0.9)
	Consultant/specialist	14 (13.2)
	Medical officer	74 (69.8)
	House officer	17 (16)
**Highest educational background**		
	Subspecialty	5 (4.7)
	Master’s degree	27 (25.5)
	Bachelor’s degree	74 (69.8)
**Work experience at MOH** ^a^ **(y; mean 9.87, SD 5.60 y)**		
	1-3	22 (20.8)
	4-5	8 (7.5)
	>5	76 (71.7)
**Work experience at current hospital (y; mean 6.25, SD 4.55 y)**		
	1-3	38 (35.8)
	4-5	16 (15.1)
	>5	52 (49.1)
**Casemix training program**		
	Attended	84 (79.2)
	Not attended	22 (20.8)
**Knowledge level (mean 76.56, SD 17.44)**		
	High	71 (67)
	Moderate	27 (25.5)
	Low	8 (7.5)

^a^MOH: Ministry of Health.

#### KMO and Bartlett Sphericity Test

The results of the KMO measure of sampling (KMO) and BTOS for these constructs. The overall KMO value was 0.859, which exceeded 0.6, and the BTOS yielded statistically significant results (aim: *P*<.001), thereby validating the suitability of the data for further analysis [93,96,105,106,108,117,127,128]. On the basis of the results of both the significant BTOS and the value of >0.6 for the KMO measure, it can be concluded that the data were suitable for the data reduction procedure [105,106,108,109,113,117].

### EFA Results

**Figure 2:**
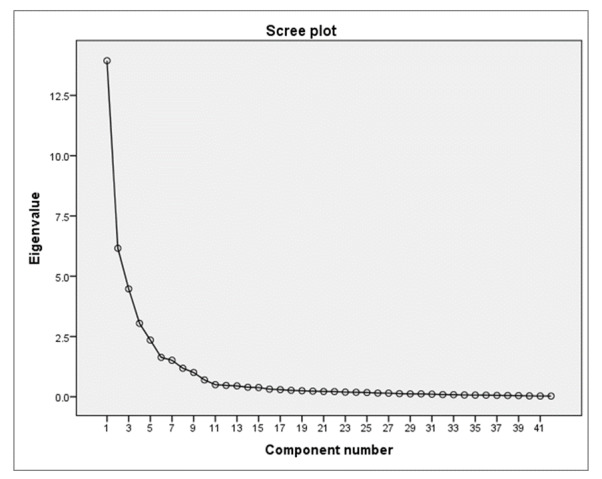
The scree plot indicating the 9 components that emerged for the constructs.

This study used EFA to analyze 106 quantitative pilot responses from the preliminary questionnaire. Some researchers have advocated for EFA to determine whether the items under investigation have distinct dimensions compared to those in previous studies [93,96,105,106,108,109,113,129]. The EFA procedure involved grouping 42 items into 9 components, each representing a distinct set of measured items. The rotated component matrix elucidated the assignment of items to specific components [105,106,108,109,113]. The scree plot provided corroboration for identifying these components, and the specific allocation of items can be found in Figure 2. This study’s findings provide valuable insights into the construct under investigation.

### Dimensions and Total Variance

The analysis indicates that 9 components with eigenvalues of >1.0 were identified. These components exhibited values ranging between 6.6 and 14.155. The variance explained by each component was as follows: 33.187% for the first component, 47.852% for the second component, 58.507% for the third component, 65.76% for the fourth component, 71.357% for the fifth component, 75.25% for the sixth component, 78.853% for the seventh component, 81.673% for the eighth component, and 84.07% for the ninth component. The TVE for this construct was 84.07%, which meets and exceeds the minimum requirement of 60%, thus making it an acceptable result [93,105,106,109,113,116,117]. If the TVE is <60%, the researcher must consider using more items to measure the constructs, which did not occur in this study. Put simply, if the TVE derived in Table 2 is <60%, the current items are insufficient for measuring the constructs [93,105,106,109,116].

**Table 2:** The number of components formed and the amount of variance^a^.

Component	Initial eigenvalues				Rotation sums of squared loadings		
	Total	Percentage of variance	Cumulative percentage	Total		Percentage of variance	Cumulative percentage
1	13.939	33.187	33.187	5.945		14.155	14.155
2	6.159	14.665	47.852	4.477		10.660	24.815
3	4.475	10.656	58.507	4.213		10.030	34.845
4	3.046	7.252	65.760	4.029		9.594	44.438
5	2.351	5.597	71.357	3.834		9.129	53.568
6	1.635	3.893	75.250	3.384		8.058	61.626
7	1.513	3.603	78.853	3.336		7.943	69.569
8	1.184	2.820	81.673	3.314		7.892	77.460
9	1.007	2.398	84.070	2.776		6.610	84.070

^a^Extraction method: principal component analysis.

### PCA Results

The PCA extraction method with varimax rotation was used to identify 9 components across all constructs, deviating from the initial conceptual framework of 8. This finding is expected to unveil novel dimensions due to its execution within a new environment [105,106,108,109,113]. The factor loading of EFA with PCA and varimax rotation was chosen based on its widespread use as an orthogonal factor rotation approach and its ability to clarify factor analysis [93,96,105,106,108,127].

The initial organizational factors (O) construct was divided into 2 components: component 4 (items O6-O9) and component 7 (items O2-O5). Another item, O1, with the statement “Organizational capacity to allocate resources for the implementation of the Casemix System in THIS context” was eliminated from the instrument as its factor loading was <0.6. The construct previously referred to as “organizational factors (O)” was renamed “organizational characteristics (ORG)” in the measurement model. The individual components within this construct were also renamed based on their specific measurements. They are consistent with the components of the related construct “organization” outlined in the HOT-Fit evaluation framework [33,34].

The remaining 7 constructs, namely, SY, IQ, SQ, PEOU, PU, ITU, and acceptance, did not form a new component and did not have any items removed. The total number of constructs remained unchanged at 8. Among the 8 constructs, 98% (41/42) of the items were retained in the measurement model. The researcher proceeded to reorganize the items into their appropriate constructs and components and then began collecting data in the field study. The PCA using varimax rotation is shown in Table 3.

Multimedia Appendix 9 presents the EFA results, indicating the number of items for each construct before and after the study. The initial 42 items were reduced to 41 (98%) following the elimination of item O1 due to a low factor loading <0.60, culminating in a final set of 41 items for the quantitative instrument.

**Table 3:** Factor loading of exploratory factor analysis with principal component analysis and varimax rotation^a^.

Item ID	Rotated component matrix^b^: component								
	1	2	3	4	5	6	7	8	9
PEOU1	—^c^	—	0.908	—	—	—	—	—	—
PEOU2	—	—	0.916	—	—	—	—	—	—
PEOU3	—	—	0.889	—	—	—	—	—	—
PEOU4	—	—	0.919	—	—	—	—	—	—
PEOU5	—	—	0.895	—	—	—	—	—	—
PU1	—	—	—	—	—	—	—	0.872	—
PU2	—	—	—	—	—	—	—	0.888	—
PU3	—	—	—	—	—	—	—	0.914	—
PU4	—	—	—	—	—	—	—	0.872	—
O1	—	—	—	0.590	—	—	—	—	—
O2	—	—	—	—	—	—	0.873	—	—
O3	—	—	—	—	—	—	0.746	—	—
O4	—	—	—	—	—	—	0.874	—	—
O5	—	—	—	—	—	—	0.861	—	—
O6	—	—	—	0.827	—	—	—	—	—
O7	—	—	—	0.864	—	—	—	—	—
O8	—	—	—	0.808	—	—	—	—	—
O9	—	—	—	0.882	—	—	—	—	—
SY1	—	—	—	—	0.826	—	—	—	—
SY2	—	—	—	—	0.821	—	—	—	—
SY3	—	—	—	—	0.836	—	—	—	—
SY4	—	—	—	—	0.853	—	—	—	—
IQ1	—	—	—	—	—	—	—	—	0.614
IQ2	—	—	—	—	—	—	—	—	0.625
IQ3	—	—	—	—	—	—	—	—	0.654
IQ4	—	—	—	—	—	—	—	—	0.689
IQ5	—	—	—	—	—	—	—	—	0.619
SQ1	—	0.754	—	—	—	—	—	—	—
SQ2	—	0.796	—	—	—	—	—	—	—
SQ3	—	0.788	—	—	—	—	—	—	—
SQ4	—	0.848	—	—	—	—	—	—	—
SQ5	—	0.830	—	—	—	—	—	—	—
ITU1	0.825	—	—	—	—	—	—	—	—
ITU2	0.792	—	—	—	—	—	—	—	—
ITU3	0.770	—	—	—	—	—	—	—	—
ITU4	0.925	—	—	—	—	—	—	—	—
ITU5	0.890	—	—	—	—	—	—	—	—
A1	—	—	—	—	—	0.676	—	—	—
A2	—	—	—	—	—	0.694	—	—	—
A3	—	—	—	—	—	0.625	—	—	—
A4	—	—	—	—	—	0.878	—	—	—
A5	—	—	—	—	—	0.833	—	—	—

^a^Extraction method: principal component analysis; rotation method: varimax with Kaiser normalization.

^b^Rotation converged in 8 iterations.

^c^Not applicable.

### The Instrument’s Internal Reliability

This study assessed the internal reliability of the retained items using the Cronbach α [130]. The Cronbach α is used to assess the internal consistency of measurement items and how well they measure the same underlying concept [93,96,105,106,108,109,128]. In this study, a Cronbach α value of ≥0.7 was necessary for assessment [121]. All studied constructs exhibited values of >0.7, indicating satisfactory internal reliability. PEOU had the highest Cronbach α at 0.969, whereas PU had the lowest at 0.914. This evaluation highlights the robustness of the measurement items in capturing the intended underlying constructs. Table 4 presents the Cronbach α score for each construct.

**Table 4:** Cronbach α for each construct.

Construct and name of component		Items	Items, N	Cronbach α (>0.7)
System quality		SY1-SY5	5	0.968
Information quality		IQ1-IQ4	4	0.950
Service quality		SQ1-SQ5	5	0.902
**Organizational factors**		O2-O9	8	0.933
	Structure	O2-O5	4	0.958
	Environment	O6-O9	4	0.919
Perceived ease of use		PEOU1-PEOU5	5	0.969
Perceived usefulness		PU1-PU4	4	0.914
Intention to use		ITU1-ITU5	5	0.949
Acceptance		A1-A5	5	0.952

## Discussion

### Overview

This study aimed to improve the quality and validity of a questionnaire used for measuring the acceptance of Casemix in the THIS setting [114,131-133]. The pilot study was conducted at one of the THIS MOH facilities in Malaysia using an SAQ based on multiple questions and adapted from previous instruments. The results showed that the SAQ is reliable and valid for assessing the critical factors and acceptance of Casemix implementation in THIS MOH hospitals in Malaysia.

### Principal Findings

#### Demographic Profiles and Knowledge Level

This study examined the demographic profile and success factors influencing the adoption and implementation of the Casemix system in the MOH of Malaysia. Gender-related outcomes in eHealth use are unclear, with some studies suggesting gender influences but few finding a correlation [134-137]. Demographic studies have had predominantly female samples; most respondents in our study were young adults aged 31 to 40, followed by those aged 21 to 30; this aligns with findings from other studies, which also report that younger individuals show greater interest in eHealth and IT [134,137-139]. In contrast, older individuals often face barriers such as limited access to devices or difficulty using them [136-138]. Respondents with significant experience in the health care industry are likely to have opinions on the Casemix system [10,43,140]. The length of service or seniority of health care workers may also impact their knowledge and acceptance of the system [141]. Education is not directly related to eHealth use, but higher levels are associated with greater knowledge and use of health IT [137,138,142,143]. Most respondents to the questionnaire (74/106, 69.8%) had a bachelor’s degree, which is the lowest requirement for medical doctors worldwide. However, this is due to most (74/106, 69.8%) being medical officers and some (17/106, 16%) being house officers [144,145]. A mean knowledge level score of 76.56% (SD 17.44%) suggests the success of training programs [14,43,146,147]. However, further enhancement in training and educational outreach is needed to ensure a consistently high level of awareness among hospital staff [57,128,148].

#### EFA Findings

The quantitative pilot study was subjected to EFA, a statistical data analysis method that helps discover the essential dimensions or interactions of a set of assessed factors. EFA helps examine correlations among variables and compute a condensed form of a set of factors known as factor loading, which shows the strength and direction of association between factors and the factors that are observable from the variables [93,105,106,109,116]. Confirmatory factor analysis evaluates and validates a proposed factor structure through EFA [93,105,106,109,116].

The primary objective of this paper is to underscore the thorough planning and confirmation of the validity assessment, reliability testing, pilot test, and EFA to evaluate the propensity of medical doctors to adopt the Casemix system in the HIS setting. Medical doctors working in a THIS context embrace the Casemix system due to its perceived significance in clinical practice, user-friendly nature, comprehensive training and support, positive impact on efficiency and productivity, and assurance of accurate data and security. To achieve acceptance, health care organizations must address these components, potentially leading to enhanced use of the Casemix system and subsequently improving patient care and outcomes.

#### KMO and Bartlett Sphericity Test

This study assessed the KMO and BTOS for all constructs. The findings showed that all constructs had KMO values of 0.859, which was >0.6, and the BTOS yielded statistically significant results (aim: *P*<.001), as supported by previous literature [93,96,105,106,108,117,127,128]. These results indicate that it was appropriate to proceed with further analysis. Subsequently, the pilot data were re-explored to identify any new components and their respective items.

The EFA procedures identified 9 components, which are now recognized as constructs, from the 42 items analyzed. This is a shift from the 8 constructs previously identified in the literature based on 2 frameworks [32,34,35,37]. The emergence of an additional construct suggests that the data may encompass previously unforeseen dimensions. This transition in the number of constructs from 8 to 9 could be attributed to changes in the demographic characteristics of the population studied, including factors such as socioeconomic level and educational attainment [93,105,106].

#### Dimensions and Total Variance

The TVE is a crucial measure that evaluates the extent to which specific factors account for variation in a dataset. It is essential in determining the accuracy of discovered components in representing the data structure. The mean TVE for all constructs was 84.07%, meeting the minimum criterion of 60% [105,106,149,150]. This high TVE indicates a good factor structure and the ability of the system to capture the major factors of acceptance of the Casemix system within the THIS context [96,116]. This enhances the model’s interpretability, especially in systems with a series of emerging technologies, such as HITS applications. Previous research supports the importance of high TVE in substantiating factor models consistently [112,151]. This study showed a substantial relationship between identified factors and parameter variability related to the Casemix system, proving the validity and accuracy of the factor model.

#### PCA Findings

This study observed 9 different slopes in the scree plot. We extracted 9 components from the data using an 8-construct model, where one of the constructs bifurcates into 2 [93,105,108,109,152]. Demographic differences such as variations in socioeconomic level, educational background, and populations may contribute new aspects to the elements that influence system acceptance, thus contributing to these PCA findings [153]. The instrument contained 42 items, with 41 (98%) having factor loadings of >0.6. Varimax rotation was selected due to its capacity to elucidate factor analysis and generate more comprehensible components [93,105,106,108,127]. The researchers divided the organizational factors construct into 2 components, component 4 and component 7, based on the specific items that each component measures. The organizational factors construct was renamed organizational characteristics in the measurement model, with component 4 renamed organizational structure and component 7 renamed organizational environment [93,105,106,108,127]. This division is similar to the HOT-Fit evaluation framework’s 2 components for the organizational factors dimension [33,35]. The other 7 constructs (SY, IQ, SQ, PEOU, PU, ITU, and acceptance) remained unchanged [30,33,35]. The new measurement model shares similarities with the integrated HOT-Fit and TAM frameworks as per the study’s conceptual framework [30,33,35].

#### Internal Consistency and Reliability

The internal consistency and reliability of the measurement items were evaluated using the Cronbach α, which is crucial in determining reliability [121,130]. In this study, the Cronbach α for PEOU was 0.914, and the internal reliability for PU was 0.969. These outcomes are in line with previous empirical works that underlined ease of use and usefulness as significantly influencing the adoption of technology [30,31,36].

### Theoretical Contributions, Limitations, and Recommendations

#### Theoretical Contributions

This study’s findings are context specific, but the principles and methodologies can serve as models for researchers in other settings. By adapting these methods, researchers in different countries can conduct similar studies tailored to their health care environments [154-156]. Identifying CSFs for health care information systems such as the Casemix system presents a universal challenge [154,157]. This study’s conceptual framework and analytical methods aid in understanding system acceptance and use in diverse contexts, which can guide researchers and policy makers worldwide in implementing and optimizing health care information systems [79,158,159].

While the specifics of the Casemix system may vary across countries, the overarching goals of better resource allocation, clinical decision-making, and quality of care are shared worldwide. This study’s findings, particularly regarding factors influencing system acceptance, are relevant to stakeholders worldwide, contributing to the broader knowledge of health care information systems and informing practices in diverse settings [154-156]. The dissemination of best practices from this study can enhance health care information systems worldwide, fostering more efficient and effective health care delivery.

Following the initial validation, reliability assessments, and EFA procedures, the instrument can be considered validated and reliable for field study. It will be used to assess the willingness of medical doctors to integrate the Casemix system into their daily practice within the THIS setting. The objective is to assist policy makers and administrators in identifying crucial aspects that impact the effective implementation of the system [160,161]. Key elements influencing physicians’ readiness to adopt the Casemix system in hospitals equipped with a comprehensive THIS environment include uninterrupted clinical support; strong leadership; and a dedicated team of case managers, nurses, and paramedical professionals [159,162]. Understanding the importance of operational information in an information system could enhance its efficiency [162-164]. In summary, the findings of this study provide reliability along with early validity evaluations in preparation for a field study that will assess critical factors and the acceptability of successful Casemix system adoption within the THIS environment.

#### Limitations

This study has some limitations. This study focused on the implementation of the Casemix system in a specific medical setting—the IT environment of Malaysia. It lacks reliable data from previous studies and literature, making this a research gap. The researchers developed a new conceptual framework based on the study population, geographical areas, and cultural differences and adopted items from validated questionnaires. The instrument’s reliability was assessed through a pilot study and EFA to prepare for the final instrument used during the field study.

This study excluded professional positions such as paramedics, medical record officers, IT officers, and finance officers due to their different job scopes and educational backgrounds. The pilot study could introduce bias or errors into the main study, potentially impacting the representativeness and generalizability of the sample. The findings are likely to be specific to the health care setting in Malaysia and may not immediately apply to other countries or health care systems with unique sociocultural, organizational, or technological features. This study’s large sample size was limited to 1 specifically chosen THIS hospital, reducing its generalizability. The quantitative data collected through the Google Form questionnaire may not offer a thorough understanding of the study participants, as some may have completed the questionnaire without fully comprehending the items. In addition, no researcher could be reached face-to-face if participants had a problem understanding the context of the questions.

#### Recommendations

This study recommends implementing the Casemix system in other industries and conducting tests on numerous individuals and sectors to enhance the findings. Future studies should incorporate comparative studies conducted in diverse cultural and organizational contexts to differentiate between universal and context-specific aspects that impact the adoption and acceptability of the system [138,165]. Expanding the target demographic to include more health care professions, such as medical record officers, paramedics, or health care IT professionals, could improve the generalizability of the results [82]. Future research should use longitudinal designs to monitor the evolution of perceptions and acceptance over an extended period, gaining a more profound understanding of the implementation dynamics of the system [166]. Comparing the findings by analyzing this study’s instrument using an alternative analysis tool would also be recommended.

### Conclusions

Conclusively, the focus of this study was to explore items based on the constructs of the system, information and SQ, organizational characteristics, PEOU, PU, ITU, and acceptance. This was to develop a quantitative instrument measuring the acceptance of the Casemix system implementation within the THIS setting. All the constructs met the Bartlett test requirements (significant with *P*<.001) and had satisfactory KMO scores (>0.6), indicating that the instrument is suitable for factor loading. EFA is crucial for developing and evaluating study instruments. In this study, EFA was based on pilot data, enhancing its practical use. By analyzing various criteria, including scree plots and item-total correlations, EFA suggested a 9-factor solution. Of 42 items, 41 (98%) were retained for the final instrument, explaining 84.07% of the TVE. Using EFA with PCA and varimax rotation, 9 components were extracted, with only 1 construct splitting into 2 components. These components, related to organizational characteristics, were identified as structure and environment. A total of 42 items were used to measure these constructs, with 41 (98%) items showing factor loadings exceeding the minimum value of 0.6. One item (O1) was removed due to a low factor loading of <0.6. The remaining items for all constructs demonstrated high Cronbach α values, indicating strong internal reliability. The results confirmed the relevance of the items for this study. The final questionnaire was validated and is considered reliable for field studies. The refinement and validation of the preliminary instrument ensured its internal consistency and validity across the sample. Further research should broaden the target population and increase the sample size for more robust findings. This study aimed to guide policy makers, health care professionals, and administrators regarding strategic service expansion and solutions to identified challenges. Worldwide, this study provides a tool for assessing the effectiveness of technology-driven solutions. Evaluating the Casemix system’s quality and usefulness is vital for making informed decisions and ensuring its long-term acceptability and sustainability in the THIS setting.

## Appendix

Multimedia Appendix 1 Operational definitions.

Multimedia Appendix 2 Content validity index definitions, formulas, and calculations.

Multimedia Appendix 3 Criterion validity by expert.

Multimedia Appendix 4 Face validity.

Multimedia Appendix 5 Inclusion and exclusion criteria.

Multimedia Appendix 6 Blank copy of quantitative instrument (questionnaire).

Multimedia Appendix 7 Participant information sheet.

Multimedia Appendix 8 Consent form.

Multimedia Appendix 9 The number of items for each construct before and after EFA.

## Abbreviations

BTOS: Bartlett test of sphericity
CSF: critical success factor
CVI: content validity index
EFA: exploratory factor analysis
HIS: hospital information system
HITS: health care information technology system
HOT-Fit: human, organization, technology-fit
IQ: information quality
ITU: intention to use
KMO: Kaiser-Meyer-Olkin
MOH: Ministry of Health
PCA: principal component analysis
PEOU: perceived ease of use
PU: perceived usefulness
SAQ: self-administered questionnaire
SQ: service quality
SY: system quality
TAM: technology acceptance model
THIS: Total Hospital Information System
TVE: total variance explained
