# Supplementary material for: Critical Success Factors and Acceptance of the Casemix System Implementation Within the Total Hospital Information System: Exploratory Factor Analysis of a Pilot Study
Source: JMIR Form Res. 2024 Oct 29;8:e56898. doi: 10.2196/56898 (PMC11558226; doi:10.2196/56898)
Supplement: Multimedia Appendix 1 [file formative_v8i1e56898_app1.pdf]

Multimedia Appendix 1: Operational Definitions

Section/ Sub-Section	Variable/Construct	Operational Definitions	References/Sources
1.1	Gender	The classification of individuals into male and female categories is based on biological and social characteristics.	[1–5]
	Age	Age refers to the respondent's age in January 2023. The categorization of individuals into age groups: Less than 25, 25-30, 31-40, 41-50, and 51-60 years old, representing different life stages and experiences. Age determines a person's survey knowledge and experience.	[3,6–9]
	Professional Role	The categorization of individuals based on the MOH-approved position or profession at the hospital: hospital directors, deputy directors, consultants/specialists, medical officers, and house officers, reflecting different levels of expertise and responsibilities. This is to measure the relationship between the occupational status of individuals with their use of HIS and/or Casemix System	[2,10–12]
	Highest Education Level	The classification of individuals based on their highest level of educational attainment: post-doctorate, doctorate, sub-specialization, master's degree, and bachelor's degree, indicating different levels of knowledge and skills.	[2,10,13,14]
	Tenure at the Ministry of Health	The classification of individuals based on their tenure working within the Ministry of Health: <3 years, 3-5 years, >5 years, reflecting different levels of familiarity	[10,15–19]

		and experience within the organization.	
	Tenure at the Current Hospital	The classification of individuals based on their duration of employment at the current hospital: <3 years, 3-5 years, >5 years, indicating different levels of familiarity with the hospital's environment and operations.	[10,15–19]
	Casemix Training Experience	The classification of individuals is based on whether they have received training on Casemix: had training experience or no training experience, indicating different levels of exposure to Casemix concepts and practices. This is also can be referred to as a history of training experience in healthcare information technology (HIT)/ hospital information system (HIS), since the Casemix system is also a part of healthcare information technology. Hence, the option of the response is either yes or no history of training.	[6,20,21]
1.2	Knowledge of Casemix	Refers to a respondent's correct answers regarding the Casemix system and HIS based on standard guidelines (Ministry of Health Malaysia 2016). The 10-likert scale will be employed which indicates 1 representing "no knowledge," 10 representing "excellent Knowledge" and the midway point 5 representing "fair knowledge". The total scores ranged from 10–100 points. The minimum score was 10; the maximum score was 100.<br><br>The classification of individuals based on their level of understanding of Casemix: below-average knowledge	[2,10,22–28]

		(score of 10-50), average knowledge (score of 51-70), and above-average knowledge (score of 71-100), representing different levels of familiarity and expertise. Below-average scores are classified as low knowledge; average scores are classified as intermediate knowledge, and above-average scores are considered high knowledge	
2.1	System Quality	The extent to which a system meets user expectations performs reliably, is free of errors, and is easy to operate and navigate. This construct refers to a respondent's critical success factors that facilitate or influence the acceptance of Casemix System implementation among medical doctors. This construct consists of a total of 4 items. The 10-Likert scale will be employed which indicates zero representing "strongly disagree," 10 representing "strongly agree" and the midway point five representing "neither agree nor disagree". The total scores ranged from 4–40 points.	[29–38]
2.2	Information Quality	Information quality consists of the accuracy, completeness, relevancy, and timeliness of the information provided by the system. This construct refers to a respondent's critical success factors that facilitate or influence the acceptance of Casemix System implementation among medical doctors. This construct consists of a total of 5 items. The 10-Likert scale will be employed which indicates zero representing "strongly disagree," 10 representing "strongly agree" and the	[29–38]

		midway point five representing "neither agree nor disagree". The total scores ranged from 5–50 points.	
2.3	Service Quality	Service quality refers to the degree of responsiveness, reliability, assurance, empathy, and tangibles provided by the service provider. This construct refers to a respondent's critical success factors that facilitate or influence the acceptance of Casemix System implementation among medical doctors. This construct consists of a total of 5 items. The 10-Likert scale will be employed which indicates zero representing "strongly disagree," 10 representing "strongly agree" and the midway point five representing "neither agree nor disagree". The total scores ranged from 5–50 points.	[29,35–42]
2.5	Perceived Ease of Use	Perceived Ease of Use refers to the degree to which a person believes that using a particular system would be free of effort. This construct refers to a respondent's critical success factors that facilitate or influence the acceptance of Casemix System implementation among medical doctors. This construct consists of a total of 5 items. The 10-Likert scale will be employed which indicates zero representing "strongly disagree," 10 representing "strongly agree" and the midway point five representing "neither agree nor disagree".	[31,43–47]

		The total scores ranged from 5–50 points.	
2.6	Perceived Usefulness	Perceived usefulness refers to the degree to which a person believes that using a particular system would enhance their job performance. This construct refers to a respondent’s critical success factors that facilitate or influence the acceptance of Casemix System implementation among medical doctors. This construct consists of a total of 4 items. The 10-Likert scale will be employed which indicates zero representing "strongly disagree," 10 representing “strongly agree” and the midway point five representing "neither agree nor disagree". The total scores ranged from 4–40 points.	[31,43–47]
2.4	Organizational Characteristics	Organizational factors refers to the collective attributes, features, and elements that define an organization, encompassing its culture, structure, leadership style, strategy, and systems. This construct refers to a respondent’s critical success factors that facilitate or influence the acceptance of Casemix System implementation among medical doctors. This construct consists of a total of 9 items. The 10-Likert scale will be employed which indicates zero representing "strongly disagree," 10 representing “strongly agree” and the	[33,35–38,48–50]

		midway point five representing "neither agree nor disagree". The total scores ranged from 9–90 points. However, this construct was then renamed as organizational characteristics and has been divided into two components, which are organizational structure and organizational environment.	
2.4	Organizational Structure	Organizational Structure refers to the formal arrangement of roles, responsibilities, and relationships within an organization, including hierarchical levels, reporting relationships, and decision-making processes. This component emerged from the organizational factors, and it refers to a respondent’s critical success factors that facilitate or influence the acceptance of Casemix System implementation among medical doctors. This component consists of a total of 4 items. The 10-Likert scale will be employed which indicates zero representing "strongly disagree," 10 representing “strongly agree” and the midway point five representing "neither agree nor disagree". The total scores ranged from 4–40 points.	[35–38,51–53]
	Organizational Environment	Organizational environment refers to the internal and external factors, such as culture, leadership, and external pressures, that influence the organization's operations and performance. This component emerged from the organizational factors, and it refers to a respondent’s critical success factors that facilitate or influence the	[33,35–38,48–50,54,55]

		acceptance of Casemix System implementation among medical doctors. This component consists of a total of 4 items. The 10-Likert scale will be employed which indicates zero representing "strongly disagree," 10 representing “strongly agree” and the midway point five representing "neither agree nor disagree". The total scores ranged from 4–40 points.	
2.7	Intention to Use	Intention to Use refers to the individual's readiness and willingness to use a system, often influenced by perceived usefulness, ease of use, and external factors. This construct refers to a respondent’s critical success factors that facilitate or influence the acceptance of Casemix System implementation among medical doctors. This construct consists of a total of 5 items. The 10-Likert scale will be employed which indicates zero representing "strongly disagree," 10 representing “strongly agree” and the midway point five representing "neither agree nor disagree". The total scores ranged from 5–50 points.	[44,46,56–59]
	Acceptance of the Casemix System	Acceptance of the Casemix System refers to the extent to which users (for this study medical doctors) perceive the Casemix system as useful, and easy to use, and are willing to adopt it within the hospital information system. This construct refers to a respondent’s critical success factors that facilitate or influence the acceptance of Casemix System implementation among medical doctors. This construct	[44,46,57–61]

		consists of a total of 5 items. The 10-Likert scale will be employed which indicates zero representing "strongly disagree," 10 representing “strongly agree” and the midway point five representing "neither agree nor disagree". The total scores ranged from 5–50 points.	

REFERENCES:

1. Alice H E, Wood W. The Origins of Sex Differences in Human Behavior: Evolved Dispositions versus Social Roles. *Am Psychol*. 1999;54(6):408-423. doi:10.7551/mitpress/2874.003.0015

2. Ali Jadoo SA, Aljunid SM, Dastan I, et al. Turkish healthcare providers’ level of knowledge, attitude and practice toward diagnosis related group system - A cross sectional study. *Malaysian J Public Heal Med*. 2016;16(1):121-128.

3. Khechine H, Lakhal S, Pascot D, Bytha A. UTAUT Model for Blended Learning: The Role of Gender and Age in the Intention to Use Webinars. *Interdiscip J e-Skills Lifelong Learn*. 2014;10:033-052. doi:10.28945/1994

4. Gefen D, Straub DW. Gender differences in the perception and use of e-mail: An extension to the technology acceptance model. *MIS Q Manag Inf Syst*. 1997;21(4):389-400. doi:10.2307/249720

5. Venkatesh V, Morris MG, Ackerman PL. A Longitudinal Field Investigation of Gender Differences in Individual Technology Adoption Decision-Making Processes. *Organ Behav Hum Decis Process*. 2000;83(1):33-60. doi:10.1006/obhd.2000.2896

6. LaMonica H, English A, Hickie I, et al. Examining Internet and eHealth Practices and Preferences: Survey Study of Australian Older Adults With Subjective Memory Complaints, Mild Cognitive Impairment, or Dementia. *J Med Internet Res*. 2017;19(10). doi:10.2196/jmir.7981

7. Hashim J, Wok S. Competence, performance and trainability of older workers of higher educational institutions in Malaysia. *Empl Relations*. 2014;36(1):82-106. doi:10.1108/ER-04-2012-0031

8. Morris MG, Venkatesh V. Age differences in technology adoption decisions: Implications for a changing work force. *Pers Psychol*. 2000;53(2):375-403. doi:10.1111/j.1744-6570.2000.tb00206.x

9. Czaja SJ, Sharit J. Age differences in attitudes toward computers. *Journals Gerontol - Ser B Psychol Sci Soc Sci*. 1998;53(5):329-340. doi:10.1093/geronb/53B.5.P329

10. Ali Jadoo SA, Sulku SN, Aljunid SM, Dastan I. Validity and Reliability Analysis of Knowledge of, Attitude toward and Practice of a Case-mix Questionnaire among Turkish Healthcare Providers. *J Heal Econ Outcomes Res*. 2014;2(1):96-107. doi:10.36469/9891

11. Meinert DB, Peterson D. Perceived importance of EMR functions and physician characteristics. *J Syst Inf Technol*. 2009;11(1):57-70. doi:10.1108/13287260910932412

12. Ketikidis P, Dimitrovski T, Lazuras L, Bath PA. Acceptance of health information technology in health professionals: An application of the revised technology acceptance model. *Health Informatics J*. 2012;18(2):124-134. doi:10.1177/1460458211435425

13. Maruf FA, Chianakwana C, Hanif S. Perception, Knowledge, and Attitude Toward Physical Activity Behavior: Implications for Participation Among Pregnant Women. *J Womens Health Phys Therap*. 2017;41(3):145-153.

14. Rixon L, Hirani SP, Cartwright M. What influences withdrawal because of rejection of telehealth - the whole systems demonstrator evaluation. *J Assist Technol*. 2013;7(4). doi:10.1108/JAT-06-2013-0017

15. Fukada M. Nursing Competency: Definition, Structure and Development. *Yonago Acta Med*. 2018;61(1):1-7. doi:https://doi.org/10.33160%2Fyam.2018.03.001

16. Alipour J, Mehdipour Y, Karimi A. Factors Affecting Acceptance of Hospital Information Systems in Public Hospitals of Zahedan University of Medical Sciences: A Cross-Sectional Study. *J Med Life*. 2019;12(4):403-410. doi:10.25122/jml-2019-0064

17. Clayton PD, Naus SP, Bowes WA, et al. Physician use of electronic medical records: issues and successes with direct data entry and physician productivity. *AMIA Annu Symp Proc*. Published online 2005:141-145.

18. Ali Jadoo SA, Aljunid SM, Nur AM, Ahmed Z, Van Dort D. Development of MY-DRG casemix pharmacy service weights in UKM Medical Centre in Malaysia. *DARU, J Pharm Sci*. 2015;23(1):1-8. doi:10.1186/s40199-014-0075-4

19. Ke Y, Kuo C, Hung C. The effects of nursing preceptorship on new nurses' competence, professional socialization, job satisfaction and retention: A systematic review. *J Adv Nurs*. 2017;73(10):2296-2305. doi:10.1111/jan.13317.

20. Reeves S, Hean S. Why we need theory to help us better understand the nature of interprofessional education, practice and care. *J Interprof Care*. 2013;27(1):1-3. doi:10.3109/13561820.2013.751293

21. Batra R, Pall AS. Adoption and Assessment of Hospital Information Systems: A Study of Hospitals in Jalandhar. *Asia-Pacific J Manag Res Innov*. 2015;11(3):205-218. doi:10.1177/2319510x15588383

22. Saizan S, Jaudin R, Nor MZM, Sukeri S. The Importance of Clinical Documentation in the MalaysianDRG Casemix System: A Sequential Explanatory Mixed-Method Study of Ministry of Health Hospitals in Malaysia. *Malaysian J Med Heal Sci*. 2021;17(1):50-56.

23. Saizan S, Jaudin R, Yaacob NM, Sukeri S. *The MalaysianDRG Casemix System: Financial Implications of Inaccurate Clinical Documentation and Coding Error*. Vol 17.; 2021.

24. Medical Development Division MOH. MalaysianDRG Findings 2017 - 2018: National Base Rate, Demographic and Quality Indicator - Key Findings. Published online 2020. https://www.moh.gov.my/moh/resources/Penerbitan/Casemix/Garis Panduan/Casemix_%0AInfographic-2017_2018_.pdf

25. Medical Development Division MOH. Casemix MalaysianDRG Way Forward. Published 2021. https://www.coursehero.com/file/162500649/2-CSMOT-way-forwardpdf/

26. Medical Development Division MOH. The MOH Casemix System Which is Called The MalaysianDRG Is Now In Its 6th Year. Published 2016. https://www.facebook.com/medicaldevelopment/posts/the-moh-casemix-system-which-is-called-the-malaysiandrg-is-now-in-its-6th-year-o/641387939355450/

27. Ali FZ. Strategic implementation of Malaysia's Casemix system for enhanced healthcare efficiency and financial sustainability. In: *9th International Casemix Conference 2024*. ; 2024:25. https://anyflip.com/kbsde/oinw/

28. Ali FZ. Diagnosis Related Group Ministry of Health Malaysia: Overview and Benefits Realization. *Casemix Introd to Begin Work*. 2022;(May).

29. DeLone WH, McLean ER. The DeLone and McLean Model of Information Systems Success: A Ten-Year Update. *J Manag Inf Syst*. 2003;19(4):9-30. doi:10.1080/07421222.2003.11045748

30. Hsiao-Hui Wang E, Chen CY. System quality, user satisfaction, and perceived net benefits of mobile broadband services. *8th Int Telecommun Soc Asia-Pacific Reg Conf*. 2011;(March 2009):1-10. http://www.econstor.eu/handle/10419/52334

31. Bamufleh D. Modelling the Acceptance and Use of Electronic Medical Records from Patients' Point of View: Evidence from Saudi Arabia. *Int J Bus Manag*. 2021;16(7):12. doi:10.5539/ijbm.v16n7p12

32. Mohammadi H. Investigating users' perspectives on e-learning: An integration of TAM and IS success model. *Comput Human Behav*. 2015;45:359-374. doi:10.1016/j.chb.2014.07.044

33. DeLone WH, McLean ER. Information systems success: The quest for the dependent variable. *Inf Syst Res*. 1992;3(1):60-95. doi:10.1287/isre.3.1.60

34. Petter S, DeLone WH, McLean E. Measuring information systems success: Models, dimensions, measures, and interrelationships. *Eur J Inf Syst*. 2008;17(3):236-263. doi:10.1057/ejis.2008.15

35. Yusof MM, Paul RJ, Stergioulas LK. Towards a Framework for Health Information Systems. *Proc Annu Hawaii Int Conf Syst Sci*. 2006;5(C):1-10. doi:10.1109/HICSS.2006.491

36. Yusof MM, Kuljis J, Papazafeiropoulou A, Stergioulas LK. An Evaluation Framework for Health Information Systems: Human, Organization and Technology-Fit Factors (HOT-Fit). *Int J Med Inform*. 2008;77(6):386-398. doi:10.1016/j.ijmedinf.2007.08.011

37. Yusof MM, Papazafeiropoulou A, Paul RJ, Stergioulas LK. Investigating Evaluation Frameworks for Health Information Systems. *Int J Med Inform*. 2008;77(6):377-385. doi:10.1016/j.ijmedinf.2007.08.004

38. Erlirianto LM, Ali AHN, Herdiyanti A. The Implementation of the Human, Organization, and Technology-Fit (HOT-Fit) Framework to Evaluate the Electronic Medical Record (EMR) System in a Hospital. In: *Procedia Computer Science*. Vol 72. Elsevier; 2015:580-587. doi:10.1016/j.procs.2015.12.166

39. Parasuraman A, Zeithaml VA, Berry LL. SERVQUAL: A multiple-item scale for measuring consumer perceptions of service quality. *J Retail*. 1988;64(1):12-40.

40. Wong WT, Huang N, Neng-Tang. The Effects of E-Learning System Service Quality and Users' Acceptance on Organizational Learning. *Int J Bus Inf*. 2011;6(2):205-224. https://www.researchgate.net/publication/268304721

41. Al-Fraihat D, Joy M, Masa'deh R, Sinclair J. Evaluating E-learning systems success: An empirical study. *Comput Human Behav*. 2020;102:67-86. doi:10.1016/j.chb.2019.08.004

42. Ahmadi H, Nilashi M, Ibrahim O. Organizational decision to adopt hospital information system: An empirical investigation in the case of Malaysian public hospitals. *Int J Med Inform*. 2015;84(3):166-188. doi:10.1016/j.ijmedinf.2014.12.004

43. Davis FD, Bagozzi RP, Warshaw PR. User Acceptance of Computer Technology: A Comparison of Two Theoretical Models. *Manage Sci*. 1989;35(8):982-1003. doi:10.1287/mnsc.35.8.982

44. Davis FD, Venkatesh V. Measuring User Acceptance of Emerging Information Technologies: An Assessment of Possible Method Biases. *Proc Annu Hawaii Int Conf Syst Sci*. 1995;4:729-736. doi:10.1109/HICSS.1995.375675

45. Davis FD. Perceived usefulness, perceived ease of use, and user acceptance of information technology. *MIS Q Manag Inf Syst*. 1989;13(3):319-339. doi:10.2307/249008

46. Venkatesh V. Technology Acceptance Model 3 and a Research Agenda on Interventions. *Decis Sci*. 2008;39(2):273-315.

47. Haderi SM Al. System Characteristic Facilitates the Acceptance of Information Technology in Middle East culture. *Int J Bus Soc Sci*. 2014;5(6):64-69. http://search.proquest.com/docview/1541291060?accountid=17193%5Cnhttp://sfx.brad.ac.uk/sfx_local?url_ver=Z39.88-2004&rft_val_fmt=info:ofi/fmt:kev:mtx:journal&genre=article&sid=ProQ:ProQ:abiglobal&atitle=System+Characteristic+Facilitates+the+Acceptance+of+

48. Boonstra A, Broekhuis M. Barriers to the acceptance of electronic medical records by physicians from systematic review to taxonomy and interventions. *BMC Health Serv Res*. 2010;10(July). doi:10.1186/1472-6963-10-231

49. Mihalas GI, Bazavan M, Farcas DD. Implementation of health information systems in Romania. *Methods Inf Med*. 2006;45(1):121-124. doi:10.1055/s-0038-1634048

50. Handayani PW, Hidayanto AN, Ayuningtyas D, Budi I. Hospital information system institutionalization processes in indonesian public, government-owned and privately owned hospitals. *Int J Med Inform*. 2016;95:17-34. doi:10.1016/j.ijmedinf.2016.08.005

51. Mintzberg H. *Structure in Fives: Designing Effective Organizations*. Prentice-Hall; 1983. doi:https://doi.org/10.2307/2393181

52. Mintzberg H. The Structuring of Organizations BT - Readings in Strategic Management. Published online 1989:322-352. https://doi.org/10.1007/978-1-349-20317-8_23

53. Sibuea GHC, Napitupulu TA, Condrobimo AR. An Evaluation of Information System Using HOT-FIT model: A Case Study of a Hospital Information System. *Proc 2017 Int*

Conf Inf Manag Technol ICIMTech 2017. 2018;2018-Janua(November):106-111. doi:10.1109/ICIMTech.2017.8273520

54. Cameron KS, Quinn RE. *Diagnosing and Changing Organizational Culture: Based on the Competing Values Framework*. 1st ed. Jossey-Bass; 2006.

55. Faber S, van Geenhuizen M, de Reuver M. eHealth adoption factors in medical hospitals: A focus on the Netherlands. *Int J Med Inform*. 2017;100:77-89. doi:10.1016/j.ijmedinf.2017.01.009

56. Helia VN, Asri VI, Kusrini E, Miranda S. Modified technology acceptance model for hospital information system evaluation - A case study. In: *MATEC Web of Conferences*. Vol 154. EDP Sciences; 2018. doi:10.1051/matecconf/201815401101

57. Kurdi B Al, Alshurideh M, Salloum SA. Investigating a theoretical framework for e-learning technology acceptance. *Int J Electr Comput Eng*. 2020;10(6):6484-6496. doi:10.11591/IJECE.V10I6.PP6484-6496

58. Venkatesh V, Davis FD. A Theoretical Extension of the Technology Acceptance Model: Four Longitudinal Field Studies. *Manage Sci*. 2000;46(2):186-204. doi:10.1287/mnsc.46.2.186.11926

59. Venkatesh V, Morris MG, Davis GB, Davis FD. User Acceptance of Information Technology: Toward A Unified View. *MIS Q*. 2003;27(3).

60. Helia VN, Asri VI, Kusrini E, Miranda S. Modified technology acceptance model for hospital information system evaluation - A case study. *MATEC Web Conf*. 2018;154:0-4. doi:10.1051/matecconf/201815401101

61. Aggelidis VP, Chatzoglou PD. Using a modified technology acceptance model in hospitals. *Int J Med Inform*. 2009;78(2):115-126. doi:10.1016/j.ijmedinf.2008.06.006
