# Supplementary material for: Critical Success Factors and Acceptance of the Casemix System Implementation Within the Total Hospital Information System: Exploratory Factor Analysis of a Pilot Study
Source: JMIR Form Res. 2024 Oct 29;8:e56898. doi: 10.2196/56898 (PMC11558226; doi:10.2196/56898)
Supplement: Multimedia Appendix 2 [file formative_v8i1e56898_app2.pdf]

Multimedia 2: Content validity index definitions, formulas and calculations.

Multimedia Appendix 2a**:** The number of experts and its implication on the acceptable cut-off score of CVI

Number of experts	Acceptable CVI values	Source of recommendation
Two experts	At least 0.80	[1]
Three to five experts	Should be 1	[2,3]
At least six experts	At least 0.83	[2,3]
Six to eight experts	At least 0.83	[4]
At least nine experts	At least 0.78	[4]

Resource: [5]

Multimedia Appendix 2b: The definition and formula of I-CVI, S-CVI/Ave, and S-CVI/UA

CVI indices	Definition	Formula
I-CVI (item-level content validity index)	The proportion of content experts giving the item a relevance rating of 3 or 4	I-CVI = (agreed item)/(number of experts)
S-CVI/Ave (scale-level content validity index based	The average of the I-CVI scores for all items on the scale or the average of proportion relevance judged by all experts. The proportion relevant is the average relevance rating by individual experts.	S-CVI/Ave = (sum of I-CVI scores)/(number of item)<br>S-CVI/Ave = (sum of proportion relevance rating)/(number of experts)
S-CVI/UA (scale-level content validity index based on the universal agreement method)	The proportion of items on the scale that achieve a relevance scale of 3 or 4 by all experts. Universal agreement (UA) score is given as 1 when the item achieved 100% experts in agreement, otherwise, the UA score is given as 0.	S-CVI/UA = (sum of UA scores)/(number of item)

Note: The definition and formula were based on the recommendations by [1–4] in Multimedia Appendix 2a.
Resource: [5]

Multimedia Appendix 2c: The relevance ratings on the item scale by two experts

Item code	Item No.	Expert 1	Expert 2	Experts in Agreement	I-CVI	UA
**Gender**	**1.1.1**	1	1	2	1	1
**Age**	**1.1.2**	1	1	2	1	1
**Hospital's Name**	**1.1.3**	1	1	2	1	1
**Professional Roles**	**1.1.4**	1	1	2	1	1
**Educational Background**	**1.1.5**	1	1	2	1	1
**Tenure at the Ministry of Health**	**1.1.6**	1	1	2	1	1
**Tenure at the Current Hospital**	**1.1.7**	1	1	2	1	1
**Casemix Training**	**1.1.8**	1	1	2	1	1
**K1**	**1.2.1**	1	1	2	1	1
**K2**	**1.2.2**	1	1	2	1	1
**K3**	**1.2.3**	1	1	2	1	1
**K4**	**1.2.4**	1	1	2	1	1
**K5**	**1.2.5**	1	1	2	1	1
**K6**	**1.2.6**	1	1	2	1	1
**K7**	**1.2.7**	1	1	2	1	1
**K8**	**1.2.8**	1	1	2	1	1
**K9**	**1.2.9**	1	1	2	1	1
**K10**	**1.2.10**	1	1	2	1	1
**PEOU1**	**2.1.1**	1	1	2	1	1
**PEOU2**	**2.1.2**	1	1	2	1	1
**PEOU3**	**2.1.3**	1	1	2	1	1
**PEOU4**	**2.1.4**	1	1	2	1	1
**PEOU5**	**2.1.5**	1	1	2	1	1
**PU1**	**2.2.1**	1	1	2	1	1
**PU2**	**2.2.2**	1	1	2	1	1
**PU3**	**2.2.3**	1	1	2	1	1
**PU4**	**2.2.4**	1	1	2	1	1
**O1**	**2.3.1**	1	1	2	1	1
**O2**	**2.3.2**	1	1	2	1	1
**O3**	**2.3.3**	1	1	2	1	1
**O4**	**2.3.4**	1	1	2	1	1
**O5**	**2.3.5**	1	1	2	1	1
**O6**	**2.3.6**	1	1	2	1	1
**O7**	**2.3.7**	1	1	2	1	1
**O8**	**2.3.8**	1	1	2	1	1
**O9**	**2.3.9**	1	1	2	1	1
**SY1**	**2.4.1**	1	1	2	1	1
**SY2**	**2.4.2**	1	1	2	1	1
**SY3**	**2.4.3**	1	1	2	1	1

**SY4**	**2.4.4**	1	1	2	1	1
**IQ1**	**2.5.1**	1	1	2	1	1
**IQ2**	**2.5.2**	1	1	2	1	1
**IQ3**	**2.5.3**	1	1	2	1	1
**IQ4**	**2.5.4**	1	1	2	1	1
**IQ5**	**2.5.5**	1	1	2	1	1
**SQ1**	**2.6.1**	1	1	2	1	1
**SQ2**	**2.6.2**	1	1	2	1	1
**SQ3**	**2.6.3**	1	1	2	1	1
**SQ4**	**2.6.4**	1	1	2	1	1
**SQ5**	**2.6.5**	1	1	2	1	1
**ITU1**	**2.7.1**	1	1	2	1	1
**ITU2**	**2.7.2**	1	1	2	1	1
**ITU3**	**2.7.3**	1	1	2	1	1
**ITU4**	**2.7.4**	1	1	2	1	1
**ITU5**	**2.7.5**	1	1	2	1	1
**A1**	**3.1**	1	1	2	1	1
**A2**	**3.2**	1	1	2	1	1
**A3**	**3.3**	1	1	2	1	1
**A4**	**3.4**	1	1	2	1	1
**A5**	**3.5**	1	1	2	1	1
				**S-CVI/Ave**	**1.00**	
**Proportion Relevance**		1	1	**S-CVI/UA**		**1.00**

**The average proportion of items judged as relevant across the two experts**	**1.00**

REFERENCES:

1. Davis LL. Instrument review: Getting the most from a panel of experts. *Appl Nurs Res*. 1992;5(4):194-197. doi:10.1016/S0897-1897(05)80008-4

2. Polit DF, Beck CT. The content validity index: are you sure you know what's being reported? Critique and recommendations 2006;29(5): *Res Nurs Health*. 2006;29(5):489–497. doi:10.1002/ nur.20147

3. Polit DF, Beck CT, Owen S V. Is the CVI an acceptable indicator of content validity? Appraisal and recommendations. *Res Nurs Health*. 2007;30(4):459–467. doi:10.1002/nur.20199

4. Lynn M. R. Determination and quantification of content validity. *Nurs Res*. 1986;35(6):382-386. http://ijoh.tums.ac.ir/index.php/ijoh/article/view/26

5. Yusoff MSB. ABC of Content Validation and Content Validity Index Calculation. *Educ Med J*. 2019;11(2):49-54. doi:10.21315/eimj2019.11.2.6
