# Supplementary material for: Critical Success Factors and Acceptance of the Casemix System Implementation Within the Total Hospital Information System: Exploratory Factor Analysis of a Pilot Study
Source: JMIR Form Res. 2024 Oct 29;8:e56898. doi: 10.2196/56898 (PMC11558226; doi:10.2196/56898)
Supplement: Multimedia Appendix 3 [file formative_v8i1e56898_app3.pdf]

UNIVERSITI KEBANGSAAN MALAYSIA

The National University of Malaysia

JABATAN KESIHATAN MASYARAKAT • *DEPARTMENT OF COMMUNITY HEALTH*

UKM.FPR JKM.600-4/4/8
5 Januari 2023

PROF. DR. [illegible]
Fakulti Perniagaan dan Pengurusan
Universiti Sultan Zainal Abidin
21300 Kuala Nerus
Terengganu

YBhg. Prof,

PERMOHONAN PERKHIDMATAN SEBAGAI PAKAR PENILAIAN SOAL SELIDIK PENYELIDIKAN TESIS PhD

Dengan segala hormatnya izinkan saya merujuk kepada perkara di atas.

2. Sukacita dimaklumkan bahawa Dr. Noor Khairiyah binti Mustafa (P115190) merupakan seorang pelajar Doktor Falsafah (Kesihatan Masyarakat), Jabatan Kesihatan Masyarakat, Fakulti Perubatan, Universiti Kebangsaan Malaysia di bawah penyelian saya. Beliau sedang menjalankan kajian bertajuk **"Critical Success Factors and The Acceptance of Casemix System Implementation in Total Hospital Information System of Ministry of Health Malaysia".**

3. Sehubungan itu, pihak fakulti berbesar hati melantik YBhg. Prof sebagai pakar menilai soal selidik bagi menentukan kesahan dan kebolehpercayaan data kuantitatif yang akan digunakan dalam penyelidikan beliau. Bersama-sama ini, dilampirkan borang soal selidik dan maklumbalas penilaian soal selidik penyelidikan tesis PhD untuk tujuan rujukan YBhg. Prof.

Saya amat berharap agar permohonan ini mendapat maklum balas yang positif dari YBhg. Prof. Perhatian dan pertimbangan dari pihak YBhg. Prof amat saya hargai.

Sekian, terima kasih.

Saya yang menjalankan amanah,

MEJ. BERSEKUTU (PA) DR. ROSZITA IBRAHIM
Jabatan Kesihatan Masyarakat
Fakulti Perubatan UKM

JABATAN KESIHATAN MASYARAKAT
Tingkat 6, Blok Praklinikal, Fakulti Perubatan UKM, 56000 Cheras, Kuala Lumpur
Tel.: +603-9145 5888/5887 Faks: +603-9145 6670 E-mel: kjjkm@ppukm.ukm.edu.my
Web:https://www.ppukm.ukm.my/jkm/en/
Mengilham Harapan, Mencipta Masa Depan • ***Inspiring Futures, Nurturing Possibilities***
www.ukm.my

Questionnaire Criterion Validation	
Title	Critical Success Factors and The Acceptance of Casemix System Implementation in Total Hospital Information System of The Ministry of Health Malaysia
Ph.D. Student	Dr. Noor Khairiyah binti Mustafa
Main Supervisor	Mejar Bersekutu (PA) Dr. Roszita binti Ibrahim
Co-Supervisors	1. Prof. Dato' Dr. Syed Mohamed Al-Junid bin Syed Junid<br>2. Prof Madya Dr. Azimatun Noor binti Aizuddin

Conceptual Framework

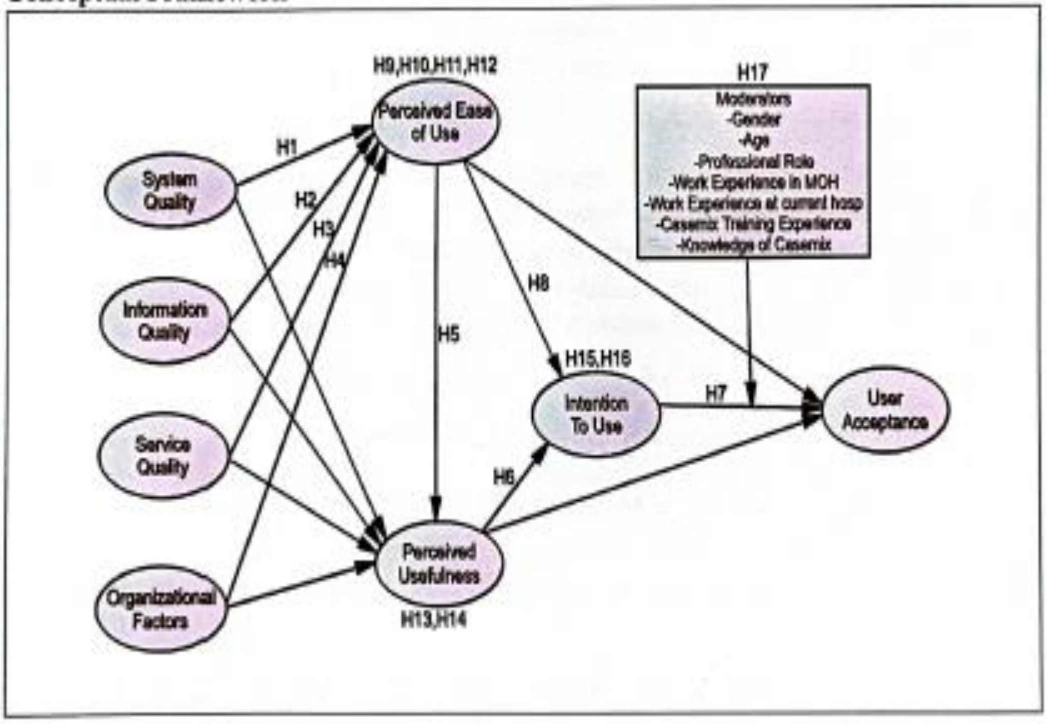

CRITERION VALIDATION OF THE QUESTIONNAIRE

Dear Experts,

This questionnaire contains eight (8) main constructs/domains with a total of 60 items. We need your judgment on the degree of match of each item. Your review should be based on the definitions, and relevant terminologies that are provided in the questionnaire. Please be as objective and constructive as possible in your review and please use the following rating scale:

<u>Degree of Match:</u>

Perfect Match - maintain the item as it is
Moderate Match - maintain the item but needs some refining
Poor Match - remove the item

QUESTIONNAIRE
(BORANG KAJI SELIDIK)

Title: Critical Success Factors and the Acceptance of Casemix System Implementation in Total HospitalInformation System of the Ministry of Health Malaysia

Tajuk: Faktor Kejayaan Kritikal Dan Penerimaan Bagi Pelaksanaan Sistem Casemix di Sistem Maklumat Hospital Menyeluruh Di Kementerian Kesihatan Malaysia

INSTRUCTIONS

ARAHAN

This questionnaire is divided into three sections which consist of **i) Personal Details, ii) The Critical Success Factors of Casemix Implementation in THIS, and iii) Outcome of the study which is the Acceptance Leveltowards Casemix System implementation in THIS setting.** The purpose of this study is to examine the critical success factors and the acceptance of Casemix System implementation in THIS facilities of the Ministryof Health (MOH) Malaysia.

Borang soalselidik ini terbahagi kepada tiga bahagian, iaitu ***i) Maklumat Peribadi, ii) Faktor Kejayaan Kritikalbagi Pelaksanaan Sistem Casemix di hospital yang dilengkapi fasiliti THIS dan iii) Hasil kajian iaitu Tahap Penerimaan terhadap Pelaksanaan Sistem Casemix di hospital yang dilengkapi fasiliti THIS.*** *Tujuan kajian ini adalah untuk mengkaji faktor-faktor kejayaan kritikal serta tahap penerimaan bagi Pelaksanaan Sistem Casemixdi hospital-hospital Kementerian Kesihatan Malaysia yang dilengkapi fasiliti THIS.*

Please indicate your most appropriate response. All information and your chosen answers will be kept confidential. This study has also received ethical approval from the Medical Research Ethics Committee from the Ministry of Health Malaysia **(NMRR-ID-22-02621-DKX),** and from Universiti Kebangsaan Malaysia **(JEP-2022- 777).** For more information or clarification, do not hesitate to contact the Principal Researcher via her email address: p115190@siswa.ukm.edu.my or you can contact her via WhatsApp at 012-6161342.

Sila tandakan jawapan pilihan anda. Semua maklumat dan jawapan pilihan anda akan dirahsiakan. Kajian ini juga telah mendapat kelulusan etika daripada Jawatankuasa Etika Penyelidikan Perubatan dari Kementerian Kesihatan Malaysia ***(NMRR-ID-22-02621-DKX)*** *dan dari Universiti Kebangsaan Malaysia* ***(JEP-2022-777).*** *Untuk maklumat lanjut atau penjelasan, jangan teragak-agak untuk menghubungi Penyelidik Utama melalui alamat e-mel beliau:* ***p115190@siswa.ukm.edu.my*** *atau anda boleh menghubunginya melalui WhatsApp di* ***012-6161342.***

DEFINITIONS OF TERMS AND ACRONYM

DEFINISI BAGI TERMA DAN AKRONIM

Here are some definitions of terms and acronyms that will be used in this questionnaire:
Berikut ialah beberapa definisi istilah dan akronim yang akan digunakan dalam soal selidik ini:

1) Casemix System: A system that **provides the healthcare industry with a consistent method of classifying types of patients, their treatment and associated costs**. It involves developing and implementing a patient classification system that groups patients according to their clinical conditions.
1) Sistem Casemix: Sistem yang ***menyediakan industri penjagaan kesihatan dengan kaedah yang konsisten bagi mengklasifikasikan jenis pesakit, rawatan mereka dan kos yang berkaitan.*** *Ia melibatkan*

pembangunan dan pelaksanaan sistem klasifikasi pesakit yang mengumpulkan pesakit mengikut keadaan klinikal mereka.

2) **Total Hospital Information System (THIS):** It is a project by the Ministry of Health (MOH) to provide a **complete ICT system** in establishing a **paperless** hospital environment to offer quality health services to the public- **an integration of clinical, administrative, and financial systems.**
2) Total Hospital Information System (THIS): Ia adalah projek Kementerian Kesihatan (KKM) dengan objektifuntuk menyediakan ***sistem ICT yang lengkap*** *dalam mewujudkan* ***persekitaran hospital tanpa kertas*** *untuk*

*menawarkan perkhidmatan kesihatan yang berkualiti kepada orang ramai—***satu integrasi sistem klinikal, pentadbiran dan kewangan.***

3) **MalaysianDRG Casemix System: A comprehensive system that records casemix data** (i.e demographic profiles, patient's encounter on arrival and admissions of patients, diagnosis, treatment, investigations and procedures, discharge numbers, patients' bed days) **from the hospitals to flow into the MOH data pool.**
3) MalaysianDRG Casemix System: ***Sistem komprehensif yang merekodkan data casemix*** *(iaitu* ***data demografik pesakit, data kedatangan dan kemasukan pesakit,*** *diagnosis, rawatan, penyiasatan dan prosedur, nombor pelepasan, hari tidur pesakit)* ***dari hospital untuk mengalir ke kumpulan data KKM.***

4) **Sistem Maklumat Rawatan Pesakit (SMRP): A comprehensive medical treatment report system linkingall hospitals in the country.** The required data is entered manually (manual/ some BHIS/IHIS hospitals) or through integration from HIS (most THIS) and will be then integrated into MalaysianDRG Casemix System.
4) Sistem Maklumat Rawatan Pesakit (SMRP): Sistem laporan rawatan perubatan yang komprehensif menghubungkan semua hospital di negara ini. *Data yang diperlukan dimasukkan secara manual (manual/ beberapa hospital BHIS/IHIS) atau melalui integrasi daripada HIS (kebanyakan hospital THIS) dan kemudiannya akan disepadukan ke dalam Sistem MalaysianDRG Casemix*

Thank you for your cooperation.
Terima kasih atas kerjasama yang telah diberikan.

Section 1 (*Bahagian 1*):

A. Demographic Profile and Duration of Service
Profil Demografi dan Pengalaman Bekerja

Please tick (√) your response in the appropriate boxes and fill in the blank.
Sila tandakan √ pada kotak jawapan pilihan anda dan isi petak kosong.

1.1.1 Gender [*Jantina*]: □ Male [*Lelaki*] ✓

□ Female [*Perempuan*]

1.1.2 Age [*Umur*]:_____year(s) [*tahun*] ✓

1.1.3 Name of Hospital [N*ama Hospital*]:

□Hospital Putrajaya

□Hospital Sultanah Nur Zahirah, Kuala Terengganu ✓

□Hospital Sultan Ismail, Johor Bahru

□Hospital Sultanah Bahiyah, Alor Setar

□Pusat Jantung Sarawak

1.1.4 Role or Position [*Peranan atau Jawatan*]:

□Hospital Director (Pengarah Hospital)

□Deputy Director (Timbalan Pengarah) ✓

□Consultant Specialist/Specialist (Pakar Perunding/Pakar)

□Medical Officer (Pegawai Perubatan)

□House Officer (Pegawai Perubatan Siswazah)

1.1.5 Education Background [*Latar belakang Pendidikan*]:

□Post-Doctorate (Pasca-Kedoktoran) ✓
□Philosphy Doctor, PhD (Ijazah Kedoktoran)
□Sub-Specialty (Sub-Kepakaran)
□Master's Degree (Ijazah Sarjana)
□Bachelor's Degree (Ijazah Sarjana Muda)

1.1.6 Duration of years in Malaysian Ministry of Health
[Tempoh perkhidmatan dalam Kementerian Kesihatan]

___________year(s) [*tahun*]

1.1.7 Duration of service in Casemix-THIS hospital
[Tempoh perkhidmatan di Hospital Casemix-THIS]

____________year(s) [*tahun*]

1.1.8 Have you ever undergone training related to the Casemix system during your service at the Ministry of Health?
[*Pernahkah anda menjalani latihan berkaitan Sistem Casemix sepanjang perkhidmatan anda di Kementerian Kesihatan?*]:
□ Yes (Ya)
□No (Tidak)

Comments from Validation Experts for Section 1A:

The demographic information are used for testing the moderation effect.

[illegible]
Profesor
Pusat Pengajian Sains Pengurusan
Fakulti Perniagaan dan Pengurusan
Universiti Sultan Zainal Abidin
Kampus Gong Badak, 21300 Kuala Nerus
Terengganu

B. Knowledge about Casemix System

Pengetahuan mengenai Sistem Casemix

Please indicate your responses based on the scale from 1-10, with 1 being 'no knowledge, 5 as 'fair knowledge' and 10 being 'Good Knowledge'.
[Sila tandakan jawapan pilihan anda mengikut skala dari 1-10, di mana 1 adalah 'tiada pengetahuan', 5 adalah 'pengetahuan yang sederhana' dan 10 adalah 'pengetahuan yang baik':

No knowledge			Fair knowledge				Good knowledge		
**1**	**2**	**3**	**4**	**5**	**6**	**7**	**8**	**9**	**10**
Tiada Pengetahuan			*Pengetahuan yang sederhana*				*Pengetahuan yang baik*		

Item No.	Item Details	Relevance			Comments
	**Knowledge about Casemix System**<br>***Pengetahuan mengenai Sistem Casemix***	**Perfect Match (maintain Item as it is)**	**Moderate Match (maintain item but needs some refining)**	**Poor Match (remove item)**	
1.2.1	Casemix system is one of the MOH's strategy to improve quality of health care in medical and health facilities in Malaysia.<br><br>*Sistem Casemix merupakan salah satu strategi KKM untuk menambahbaik kualiti penjagaan kesihatan di fasiliti perubatan dan kesihatan di Malaysia.*	✓			change the score to interval scale.
1.2.2	The main objective of Casemix System is meant for calculating the costs which has been spent for each Diagnosis Related Group (DRG) or Main diagnosis Category (MDC).<br><br>*Objektif utama pelaksanaan Sistem Casemix adalah untuk mengira kos yang dibelanjakan ke atas satu satu Diagnosis Related Group (DRG) or Main diagnosis Category (MDC)*	✓			"

The score "true" and "false" is not preffered to measure the extent of knowledge.

[redacted]
Profesor
Pusat Pengajian Sains Pengurusan
Fakulti Perniagaan dan Pengurusan
Universiti Sultan Zainal Abidin
Kampus Gong Badak, 21300 Kuala Nerus
Terengganu

1.2.3	Casemix System is involving and evaluating both clinical and costing data.<br><br>*Sistem Casemix melibatkan dan menilai kedua-dua data klinikal dan kewangan.*	✓			score change to interval scale.
1.2.4	The Casemix System is involving healthcare workers from various positions and disciplines.<br><br>*Sistem Casemix melibatkan kakitangan kesihatan daripada pelbagai jawatan dan disiplin*	✓			"
1.2.5	The implementation of the Casemix system that contains clinical data will involve demographic profiles, diagnosis, treatment, investigations and procedures.<br><br>*Pelaksanaan Sistem Casemix yang mengandungi data klinikal akan melibatkan data demografik pesakit, diagnosis, rawatan, ujian-ujian dan prosedur*	✓			"
1.2.6	The implementation of the Casemix system that contains financial data will involve space areas, annual emolument, assets, medical/health/dental supplies and medical aid supports.<br><br>*Pelaksanaan Sistem Casemix yang mengandungi data kewangan pula akan melibatkan data keluasan kawasan, emolumen (gaji) tahunan aset, bekalan pergigian/kesihatan/perubatan dan sokongan bantuan perubatan.*	✓			"
1.2.7	The implementation of Casemix System takes into account the demand and level of user's satisfaction with the health services/systems provided.<br><br>*Pelaksanaan Sistem Casemix mengambil kira keperluan, kehendak dan tahap kepuasan pengguna dengan perkhidmatan/sistem kesihatan yang disediakan*	✓			"
1.2.8	The implementation of the Casemix System means that healthcare staff provide accurate primary diagnosis, complete secondary diagnosis and complete list of procedures/ treatment/ investigations.<br><br>*Pelaksanaan Sistem Casemix bermaksud kakitangan kesihatan menyediakan diagnosis utama yang tepat, diagnosis-diagnosis lain yang lengkap dan prosedur/rawatan/ujian yang lengkap.*	✓			"
1.2.9	With the implementation of Casemix System in Total Hospital Information System (THIS), each doctor, paramedics and allied health professional is responsible for preparing accurate and complete diagnosis and procedures for acute cases, chronic cases, cases of pregnant women, and children in an integrated manner/system.	✓			"

Guna skala 1 — 10 untuk ukur
sejauh mana dia tahu atau tidak
tahu tentang sesuatu atau boleh ukur
sejauh mana dia setuju atau tidak setuju.

	Dengan Pelaksanaan Sistem Casemix di Total Hospital Information System (THIS)m, setiap doktor, paramedik dan kakitangan sokongan klinikal bertanggungjawab untuk menyediakan diagnosis dan prosedur/rawatan/ujian yang tepat dan lengkap bagi kes akut, kronik, kes ibu mengandung dan kanak-kanak dalam sistem bersepadu				
1.2.10	Monitoring and evaluation are done through indicators specially prepared for Casemix system set by the Ministry of Health Malaysia through the application of MalaysianDRG Casemix System.<br><br>*Pemantauan dan penilaian dilakukan melalui indikator-indikator yang telah disediakan khas untuk Sistem Casemix oleh pihak Kementerian Kesihatan Malaysia melalui aplikasi MalaysianDRG Casemix System.*	✓			

Other Comments from Validation Experts for Section 1B:

For measuring knowledge, it is not proper to use True or false. Need to measure how much the respondents know in a scale between 1 to 10.

So, they can assess their knowledge on subject matter based on how much they agree or disagree with the given statement.

⇒ Knowledge can also be measured based on how much they know in a scale between 1 (zero knowledge) to 10 (perfect knowledge). The score in between reflect how much the respondents know about the issue at hand.

PROF. DR. [illegible] BIN AWANG
Profesor
Pusat Pengajian Sains Pengurusan
Fakulti Perniagaan dan Pengurusan
Universiti Sultan Zainal Abidin
Kampus Gong Badak, 21300 Kuala Nerus,
Terengganu

Section 2: Critical Success Factors of Casemix System Implementation in THIS Setting
[Bahagian 2: Faktor Kejayaan Kritikal yang menyumbang kepada Implementasi Sistem Casemix dalam THIS]

This section will be divided into three sub-sections to assess the critical success factors of Casemix System Implementation in Total Hospital Information System (HIS) Setting comprising of Human, Organizational and Technological Factors in your hospital.
[Bahagian ini akan dibahagikan kepada tiga sub-bahagian untuk menilai faktor kejayaan kritikal Pelaksanaan Sistem Casemix dalam Total Hospital Information System (HIS)].

Please indicate your responses based on the scale from 1-10, with 1 being 'strongly disagree', 5 as 'neither disagree or agree'(neutral) and 10 being 'strongly agree'.
[Sila tandakan jawapan pilihan anda mengikut skala dari 1-10, di mana 1 adalah 'sangat tidak setuju', 5 adalah 'neutral' dan 10 adalah 'sangat setuju']:

Strongly disagree — Neither agree/disagree — Strongly agree

1	2	3	4	5	6	7	8	9	10

Sangat tidak setuju — neutral — *Sangat setuju*

**Factor/ Construct** ***Faktor Konstruk***	**Item No. No. Item**	**Item Details** ***Butiran Item***	**Relevance**			**Comments**
			**Perfect Match (maintain Item as it is)**	**Moderate Match (maintain item but needs some refining)**	**Poor Match (remove item)**	
**Perceived Ease of Use** ***Dirasakan Mudah untuk Digunakan***	2.1.1	It is easy to find the information I needed from THIS and/or MalaysianDRG Casemix System. *Mudah untuk mencari maklumat yang saya perlukan daripada THIS dan/atau MalaysianDRGCasemix System.*	✓			
	2.1.2	Using Casemix System with THIS is easy in all itssteps. *Menggunakan dan mengadaptasi Sistem Casemix dengan THIS adalah mudah dalam semua langkahnya.*	✓			
	2.1.3	I find it easy and flexible to get the THIS to do whatI want for Casemix purposes. *Saya rasa mudah dan fleksibel untuk THISmelakukan apa yang saya kehendaki bagi tujuan Casemix.*	✓			
	2.1.4	It is easy to learn Casemix documentation with THIS support available.	✓			

		Sangat mudah untuk mem[illegible]ajari penulisan dokumentasi klinikal Casemix dengan sokongan perkhidmatan THIS yang tersedia.				
	2.1.5	My performance at work has improved as I amusing THIS and the Casemix System.<br><br>*Prestasi saya di tempat kerja telah bertambah baik kerana saya menggunakan THIS dan mengadaptasi Sistem Casemix.*	✓			
**Perceived Usefulness**<br><br>***Dirasakan Berguna***	2.2.1	I am satisfied with the implementation of theCasemix System in THIS environment.<br><br>*Saya berpuas hati dengan pelaksanaan SistemCasemix dalam persekitaran THIS.*	✓			
	2.2.2	The use of the Casemix System in THISenvironment has facilitated my job operations.<br><br>Penggunaan Sistem Casemix dalam persekitaranTHIS telah memudahkan operasi kerja saya.	✓			
	2.2.3	Casemix System adoption in THIS context results in more success in achieving job objectives.<br><br>*Penerimaan Sistem Casemix dalam konteks THIS menghasilkan lebih banyak kejayaan dalam mencapai objektif pekerjaan.*	✓			
	2.2.4	I find the integration of THIS and/or SMRP and/or MalaysianDRG Casemix System useful in my job.<br><br>*Saya mendapati integrasi THIS dan/atau SMRP dan/atau MalaysianDRG Casemix System bergunadalam tugas saya.*	✓			
**Organizational Factors**	2.3.1	Organizational competency to provide the resources for the implementation of the Casemix System is required in THIS setting.<br><br>*Kecekapan organisasi untuk menyediakan sumber untuk pelaksanaan Sistem Casemix diperlukan dalam tetapan THIS*	✓			
	2.3.2	My employer and my head of department is supportive to me for my work involving THIS andCasemix.<br><br>Majikan dan ketua jabatan saya menyokong saya menyokong saya untuk kerja saya yang melibatkanTHIS dan Casemix.	✓			
	2.3.3	Casemix System adoption in THIS settings is supported by Ministry of Health, State Health Department and the hospital top management.<br><br>*Pelaksanaan Sistem Casemix dalam tetapan THISdisokong*	✓			

		oleh Kementeria[illegible]esihatan Malaysia, Jabatan Kesihatan Negeri dan pengurusantertinggi hospital.				
	2.3.4	Hospital top management is responsible to providetraining for Casemix and THIS.<br><br>*Pengurusan tertinggi hospital bertanggungjawab menyediakan latihan untuk Sistem Casemix dan THIS.*	✓			
	2.3.5	Series of training on the clinical documentation and costing module according to the stipulatedguidelines prepared by the Ministry of Health Malaysia are adequate.<br><br>*Siri latihan dokumentasi klinikal dan modulkewangan mengikut garis panduan yangditetapkan yang disediakan oleh Kementerian Kesihatan Malaysia adalah mencukupi.*	✓			
	2.3.6	Organizational competency leads to the easiness ofCasemix System adoption in THIS context.<br><br>*Kecekapan organisasi membawa kepada kemudahan penggunaan Sistem Casemix dalam konteks THIS.*	✓			
	2.3.7	Organizational competency leads to the usefulnessof Casemix System adoption in THIS context.<br><br>*Kecekapan organisasi membawa kepada kegunaan penggunaan Sistem Casemix dalam konteks THIS.*	✓			
	2.3.8	The service providers of THIS and/or MalaysianDRG Casemix System adequately provide training to the users.<br><br>Pembekal perkhidmatan THIS dan/atau MalaysianDRG Casemix System menyediakanlatihan secukupnya kepada pengguna.	✓			
	2.3.9	Adequate technical/application support is providedfor Casemix System implementation in THIS context.<br><br>*Sokongan teknikal/aplikasi yang mencukupi disediakan untuk pelaksanaan Sistem Casemix dalam konteks THIS.*	✓			
**System Quality**<br><br>***Kualiti Sistem***	2.4.1	The THIS and/or MalaysianDRG Casemix Systemare always available.<br><br>*THIS dan/atau MalaysianDRG Casemix Systemsentiasa tersedia.*	✓			
	2.4.2	The THIS and/or MalaysianDRG Casemix Systemare user-friendly.<br><br>*THIS dan/atau MalaysianDRG Casemix Systemadalah mesra pengguna.*	✓			

	2.4.3	interaction between users and the system.<br><br>*THIS dan/atau MalaysianDRG Casemix Systemmenyediakan interaksi antara pengguna dan sistem.*	✓			
	2.4.4	The THIS and/or MalaysianDRG Casemix Systemprovide high-speed information access.<br><br>*THIS dan/atau MalaysianDRG Casemix System menyediakan akses maklumat berkelajuan tinggi.*	✓			
**Information Quality**<br><br>***Kualiti Maklumat***	2.5.1	The information required for the Casemix systemgenerated by THIS is accurate and correct.<br><br>*Maklumat yang diperlukan untuk Sistem Casemixdijana oleh THIS adalah tepat dan betul.*	✓			
	2.5.2	The information generated by the THIS is useful for Casemix purposes by providing complete information.<br><br>*Maklumat yang dijana oleh THIS berguna untuk tujuan Casemix dengan menyediakan maklumatlengkap*	✓			
	2.5.3	The THIS generates information for Casemixpurposes in a timely manner.<br><br>*THIS menjana maklumat untuk tujuan Casemixtepat pada masanya.*	✓			
	2.5.4	The information needed for Casemix is availableall the time with THIS.<br><br>*Maklumat yang diperlukan untuk Casemix tersediasepanjang masa dengan THIS.*	✓			
	2.5.5	I trust the information output of the THIS and/or MalaysianDRG Casemix System.<br><br>*Saya mempercayai output maklumat bagi SistemTHIS dan/atau Casemix.*	✓			
**Service Quality**<br><br>***Kualiti Perkhidmatan***	2.6.1	The THIS can be relied on to provide information as and when needed for Casemix purposes.<br><br>*THIS boleh dipercayai untuk memberikan maklumat apabila diperlukan untuk tujuanCasemix.*	✓			
	2.6.2	The vendor support services of the THIS and/or SMRP and/or MalaysianDRG Casemix System provide sufficient technical assistance.<br><br>*Penyedia Sistem Casemix THIS dan/atau SMRP dan/atau MalaysianDRG menyediakan bantuan teknikal yang mencukupi.*	✓			

	2.6.3	[illegible] promise to dosomething by a specific time, they will.<br><br>*Jika perkhidmatan sokongan berjanji untukmelakukan sesuatu pada masa tertentu, mereka akan melakukannya.*	✓				
	2.6.4	The output from the integration of THIS and/or SMRP and/or MalaysianDRG Casemix system completes the Casemix workflow.<br><br>*Output daripada integrasi THIS dan/atau SMRP dan/atau MalaysianDRG Casemix System* melengkapkan proses kerja Casemix.	✓				
	2.6.5	The overall infrastructure in place is adequate tosupport THIS services and Casemix System.<br><br>*Infrastruktur keseluruhan yang disediakan adalahmencukupi untuk menyokong THIS dan Sistem Casemix.*	✓				
**Intention to Use**<br><br>***Niat untuk Menggunakan***	2.7.1	I enjoy and motivated working in a THIS hospitalwhere the Casemix System is being implemented.<br><br>*Saya suka dan bermotivasi untuk bekerja di hospital yang dilengkapi dengan THIS di manaSistem Casemix sedang dilaksanakan.*	✓				
	2.7.2	I think Casemix System with THIS providedservice implementation is a good idea.<br><br>*Saya rasa pelaksanaan Sistem Casemix dengan perkhidmatan yang disediakan THIS adalah idea yang baik.*	✓				
	2.7.3	I believe the hospital staff is receptive to theimplementation of the Casemix system.<br><br>*Saya percaya kakitangan hospital menerimapelaksanaan Sistem Casemix.*	✓				
	2.7.4	I agree with the idea of implementing Casemix System and extending it to other THIS hospitals.<br><br>*Saya bersetuju dengan idea untuk melaksanakanSistem Casemix dan memanjangkannya ke hospital yang dilengkapi dengan THIS yang lain*	✓				
	2.7.5	I recommend using THIS to support CasemixSystem implementation.<br><br>*Saya mengesyorkan penggunaan THIS bagimenyokong pelaksanaan Sistem Casemix.*	✓				

Other Comments from Validation Experts for Section 2:

The item statements are simple and easy to understand by the respondents.

[redacted]

Profesor
Pusat Pengajian Sains Pengurusan
Fakulti Perniagaan dan Pengurusan
Universiti Sultan Zainal Abidin
Kampus Gong Badak, 21300 Kuala Nerus,
Terengganu

Section 3 Outcome
[Bahagian 3: Hasil]

Please indicate your responses based on the scale from 1-10, with 1 being 'strongly disagree', 5 as 'neither disagree or agree'(neutral) and 10 being 'strongly agree'.
[Sila tandakan jawapan pilihan anda mengikut skala dari 1-10, di mana 1 adalah 'sangat tidak setuju', 5 adalah 'neutral' dan 10 adalah 'sangat setuju']:

Strongly disagree — Neither agree/disagree — Strongly agree

1	2	3	4	5	6	7	8	9	10

Sangat tidak setuju — neutral — *Sangat setuju*

Factor/ Construct<br>*Faktor Konstruk*	Item No.<br>*No. Item*	Item Details<br>*Butiran Item*	Relevance			Comments
			Perfect Match (maintain Item as it is)	Moderate Match (maintain item but needs some refining)	Poor Match (remove item)	
USER ACCEPTANCE<br><br>*PENERIMAAN PENGGUNA*	3.1	Casemix System implementation with THIS setting facilitates easy access to patient information.<br><br>*Pelaksanaan Sistem Casemix dengan tetapanTHIS memudahkan akses mudah kepada maklumat pesakit.*	✓			
	3.2	Casemix System adoption with THIS providedservice enables me to accomplish tasks more efficiently and increase my quality of work.<br><br>*Pelaksanaan Sistem Casemix dalam tetapan THISmembolehkan saya menyelesaikan tugas dengan lebih cekap dan meningkatkan kualiti kerja saya.*	✓			
	3.3	The implementation of the Casemix system inTHIS setting contributes to more accurate andcomplete diagnosis and procedures.<br><br>Pelaksanaan Sistem Casemix dalam tetapan	✓			

		THISmenyumbang kepada diagnosis dan prosedur yanglebih tepat dan lengkap.				
	3.4	**The integration of Casemix System and THIS willhelp overcome the limitations of the paper-based system.**<br><br>*Penyepaduan/Integrasi MalaysianDRG Casemix System dan/atau THIS akan membantu mengatasibatasan sistem berasaskan kertas.*	✓			
	3.5	**Overall, I am satisfied with the Casemix Systemimplementation in THIS setting.**<br><br>*Secara keseluruhannya, saya berpuas hati denganpelaksanaan Sistem Casemix dalam tetapan THIS.*	✓			

Other Comments from Validation Experts for Section 3:

The item statements are straight forward and easy to understand. No ambigous statement or misleading statement.

Language used	English<br>Correct
	Bahasa Melayu<br>Correct
Comprehensibility	✓
Appropriateness	✓

Validator's name:	[redacted]	
Contact Number:	[redacted]	
E-mail Address:	[redacted]	
Validator's signature ~~and stamp~~:		Date: [redacted]

[redacted]
Profesor
Pusat Pengajian Sains Pengurusan
Fakulti Perniagaan dan Pengurusan
Universiti Sultan Zainal Abidin
Kampus Gong Badak, 21300 Kuala Nerus,
Terengganu
