# Supplementary material for: Critical Success Factors and Acceptance of the Casemix System Implementation Within the Total Hospital Information System: Exploratory Factor Analysis of a Pilot Study
Source: JMIR Form Res. 2024 Oct 29;8:e56898. doi: 10.2196/56898 (PMC11558226; doi:10.2196/56898)
Supplement: Multimedia Appendix 4 [file formative_v8i1e56898_app4.pdf]

# Multimedia Appendix 4: Face Validity of the Questionnaire for CSF and Acceptance of Casemix System within THIS in Malaysia

| Timestamp | Email Address<br>*Alamat Emel* | Language<br>*Bahasa* | Do you agree to participate in this study?<br>*Adakah anda bersetuju untuk menyertai kajian ini?* | 1.1.1 Gender<br>*Jantina* | 1.1.2 Age (in years)<br>*Umur (tahun )* | 1.1.3 Hospital | 1.1.4 Professional Role or Position<br>*Jawatan* | 1.1.5 Education Background<br>*Latar belakang Pendidikan* | 1.1.6 Tenure in Ministry of Health Malaysia (in years)<br>*Pengalaman kerja di KKM (dalam tahun)* | 1.1.7 Tenure at current hospital (in years)<br>*Pengalaman Kerja di hospital semasa (dalam tahun)* | 1.1.8 Have you ever undergone training related to the Casemix system during your service at the Ministry of Health?<br>*Pernahkan anda menyertai apa-apa latihan berkaitan Sistem Casemix sepanjang perkhidmatan anda di KKM* | The language that has been used (i,e simple, easy to understand, hard to understand, too much medical jargon etc).<br>*Penggunaan bahasa yang telah digunakan dalam soal selidik (contoh: mudah, mudah difahami, sukar difahami, terlalu banyak jargon perubatan dll)* | Comprehensibility of instructions, questions and answer choices.<br>*Kefahaman terhadap arahan, soalan dan pilihan jawapan* | Appropriateness of instructions, questions and answer choices<br>*Kesesuaian arahan, soalan dan pilihan jawapan* | Overall comment (if any)<br>*Ulasan keseluruhan (jika ada)* |
|---|---|---|---|---|---|---|---|---|---|---|---|---|---|---|---|
| 3/9/2023 2:47:07 PM | [redacted] | Bahasa Melayu | Saya bersetuju. | Perempuan | [redacted] | Hospital Putrajaya | Pegawai Perubatan Siswazah | Ijazah Sarjana Muda | 1 | 1 | Ya | mudah difahami | Arahan, soalan dan pilihan jawapan mudah difahami. Arahan dan Soalan yang jelas untuk difahamkan. Pilihan jawapan yang banyak dan mudah untuk dipilih. Soalan yang disampaikan menepati objektif tajuk. | Arahan, soalan dan pilihan jawapan yang sesuai mengikut tajuk perbincangan. | Secara keseluruhan questionnaire sangat berkesan dan mudah difahami. Objektif yg ingin diterangkan juga dapat difahami dengan jelas. |
| 3/10/2023 9:28:08 PM | [redacted] | English | Yes, I agree. | Female | [redacted] | Hospital Putrajaya | Medical Officer | Bachelor's Degree | 6 | 1 | No | Simple and easy to understand | Overall it is comprehensive.<br>Using the scale scoring to rate answers is really helpful. | Yes, appropriate. The questions are related to the study topic | This is a good study to reveal acceptance towards casemix and factors that can contribute to its success |
| 3/13/2023 10:27:40 AM | [redacted] | Bahasa Melayu | Saya bersetuju. | Lelaki | [redacted] | Hospital Putrajaya | Pegawai Perubatan | Ijazah Sarjana Muda | 6 | 3 | Ya | Bahasa mudah difahami, terdapat sedikit penggunaan perkataan jargon perubatan | Faham seluruhnya | Sesuai dengan soalan | Kaji selidik yang bagus dan mudah difahami serta mencapai objektif |
| 3/13/2023 10:34:16 AM | [redacted] | English | Yes, I agree. | Male | [redacted] | Hospital Putrajaya | Medical Officer | Bachelor's Degree | 5 | 5 | Yes | English is easy to understand | Good | Good | Ok. A good study to investigate something that is rarely being investigated |
| 3/15/2023 10:35:52 AM | [redacted] | Bahasa Melayu | Saya bersetuju. | Perempuan | [redacted] | Hospital Putrajaya | Pakar | Ijazah Sarjana | 17 | 3 | Ya | Penggunaan Bahasa Melayu yang mudah difahami | Mudah mehami setiap arahan, soalan dan pilihan jawapan | Sesuai | Tiada |
| 3/18/2023 9:24:16 AM | [redacted] | English | Yes, I agree. | Male | [redacted] | Hospital Putrajaya | Medical Officer | Bachelor's Degree | 10 | 5 | No | Understand well | Well organized | Clear | Good initiative |
| 3/18/2023 10:34:47 AM | [redacted] | English | Yes, I agree. | Male | [redacted] | Hospital Putrajaya | Medical Officer | Master's Degree or equivalent | 16 | 1 | Yes | Slightly difficult especially the technical jargons | No major issues | No major issues | Easy to comprehend |
| 3/20/2023 11:06:47 AM | [redacted] | English | Yes, I agree. | Female | [redacted] | Hospital Putrajaya | Medical Officer | Master's Degree or equivalent | 10 | 5 | Yes | Yes.. there are some language which are lengthy and I have sent the feedback to the investigator | Easy to understand, some minor grammatical error which has been sent a feedback to the investigator | Very appropriate | A very well done questionnaire, just needs some changes in language grammar and length.. Feedback sent to the PI |
| 3/20/2023 8:25:47 PM | [redacted] | English | Yes, I agree. | Female | [redacted] | Hospital Putrajaya | House Officer | Bachelor's Degree | 1 | 1 | Yes | Understand well and the language used easy to understand | It is comprehensive. I am able to undertand the instructions, questions and answer choices well | the instructions, questions and answer choices are appropriate | Well done and all the best to the investigators |
| 3/21/2023 10:29:19 AM | [redacted] | English | Yes, I agree. | Female | [redacted] | Hospital Putrajaya | Specialist | Master's Degree or equivalent | 19 | 10 | Yes | Simple and the language is easy to understand | Comprehensive | Appropriate | Well-prepared questionnaire and study |
| 3/21/2023 12:24:08 AM | [redacted] | Bahasa Melayu | Saya bersetuju. | Lelaki | [redacted] | Hospital Putrajaya | Timbalan Pengarah | Ijazah Sarjana | 12 | 12 | Ya | Bahasa yang mudah difahami. Tiada isu | Mudah difahami kehendak soalan, arahan | Sesuai | Sebuah kaji selidik dan kajian yang sangat bagus. |

Remarks:

This private information is hidden to preserve the anonymity of the respondents.
