# Supplementary figures and images for: Critical Success Factors and Acceptance of the Casemix System Implementation Within the Total Hospital Information System: Exploratory Factor Analysis of a Pilot Study
Source: JMIR Form Res. 2024 Oct 29;8:e56898. doi: 10.2196/56898 (PMC11558226; doi:10.2196/56898)

## Multimedia Appendix 5: Inclusion and Exclusion Criteria

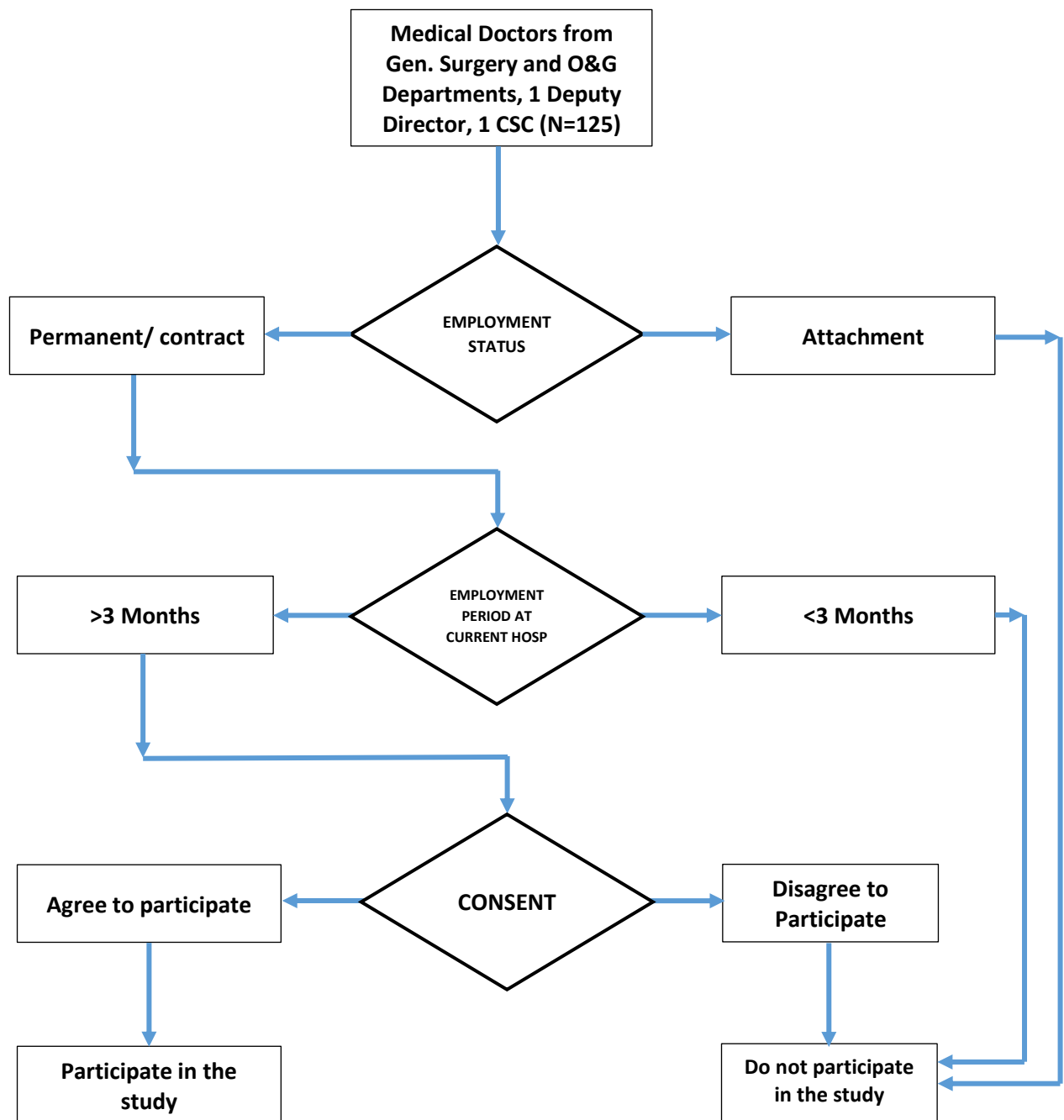

Supplement: Multimedia Appendix 5 [file formative_v8i1e56898_app5.pdf]
