# Supplementary material for: Critical Success Factors and Acceptance of the Casemix System Implementation Within the Total Hospital Information System: Exploratory Factor Analysis of a Pilot Study
Source: JMIR Form Res. 2024 Oct 29;8:e56898. doi: 10.2196/56898 (PMC11558226; doi:10.2196/56898)
Supplement: Multimedia Appendix 6 [file formative_v8i1e56898_app6.pdf]

QUESTIONNAIRE
(BORANG KAJI SELIDIK)

Title: Critical Success Factors and the Acceptance of Casemix System Implementation in Total Hospital Information System of The Ministry of Health Malaysia

Tajuk: Faktor Kejayaan Kritikal Dan Penerimaan Bagi Pelaksanaan Sistem Casemix di Sistem Maklumat Hospital Menyeluruh Di Kementerian Kesihatan Malaysia

INSTRUCTIONS
ARAHAN

This questionnaire is divided into three sections which consists of **i) Personal Details, ii) The Critical Success Factors of Casemix Implementation in THIS and iii) Outcome of the study which is the Acceptance Level towards Casemix System implementation in THIS setting.** The purpose of this study is to examine the critical success factors and the acceptance of casemix System implementation in THIS facilities of the Ministry of Health (MoH) Malaysia among medical doctors in the hospital levels including the top management (medical doctors) as well as the clinicians.

*Borang soalselidik ini terbahagi kepada tiga bahagian, iaitu **i) Maklumat Peribadi, ii) Faktor Kejayaan Kritikal bagi Pelaksanaan Sistem Casemix di hospital yang dilengkapi fasiliti THIS dan iii) Hasil kajian iaitu Tahap Penerimaan terhadap Pelaksanaan Sistem Casemix di hospital yang dilengkapi fasiliti THIS.** Tujuan kajian ini adalah untuk mengkaji faktor-faktor kejayaan kritikal serta tahap penerimaan bagi Pelaksanaan Sistem Casemix di hospital-hospital Kementerian Kesihatan Malaysia yang dilengkapi fasiliti THIS di kalangan para doctor di peringkat hospital yang terdiri daripada pengurusan tertinggi (dari kalangan doctor perubatan) serta para doktor klinikal.*

Please indicate your most appropriate response. All information and your chosen answers will be kept confidential. This study has also received ethical approval from the Medical Research Ethics Committee from the Ministry of Health Malaysia (NMRR-ID-22-02621-DKX) and from Universiti Kebangsaan Malaysia (JEP-2022- 777). For more information or clarification, do not hesitate to contact the Principal Researcher via her email address: p115190@siswa.ukm.edu.my or you can contact her via WhatsApp at 012-6161342.

*Sila tandakan jawapan pilihan anda. Semua maklumat dan jawapan pilihan anda akan dirahsiakan. Kajian ini juga telah mendapat kelulusan etika daripada Jawatankuasa Etika Penyelidikan Perubatan dari Kementerian Kesihatan Malaysia **(NMRR-ID-22-02621-DKX)** dan dari Universiti Kebangsaan Malaysia **(JEP-2022-777)**. Untuk maklumat lanjut atau penjelasan, jangan teragak-agak untuk menghubungi Penyelidik Utama melalui alamat e-mel beliau: **p115190@siswa.ukm.edu.my** atau anda boleh menghubunginya melalui WhatsApp di **012-6161342.***

DEFINITIONS OF TERMS AND ACRONYM
DEFINISI BAGI TERMA DAN AKRONIM

Here are some definitions of terms and acronyms that will be used in this questionnaire:
Berikut ialah beberapa definisi istilah dan akronim yang akan digunakan dalam soal selidik ini:

1) Casemix System: A system that **provides the healthcare industry with a consistent method of classifying types of patients, their treatment and associated costs**. It involves developing and implementing a patient classification system that groups patients according to their clinical conditions.
1) Sistem Casemix: *Sistem yang **menyediakan industri penjagaan kesihatan dengan kaedah yang konsisten bagi mengklasifikasikan jenis pesakit, rawatan mereka dan kos yang berkaitan**. Ia melibatkan pembangunan dan pelaksanaan sistem klasifikasi pesakit yang mengumpulkan pesakit mengikut keadaan klinikal mereka.*

2) **Total Hospital Information System (THIS):** It is a project by the Ministry of Health (MOH) with the objective to provide a **complete ICT system** in establishing a **paperless** hospital environment in order to offer quality health services to the public- **an integration of clinical, administrative and financial systems.**
*2) Total Hospital Information System (THIS): Ia adalah projek Kementerian Kesihatan (KKM) dengan objektif untuk menyediakan **sistem ICT yang lengkap** dalam mewujudkan **persekitaran hospital tanpa kertas** untuk*

*menawarkan perkhidmatan kesihatan yang berkualiti kepada orang ramai—**satu integrasi sistem klinikal, pentadbiran dan kewangan**.*

<u>*3)*</u> **<u>MalaysianDRG Casemix System:</u> A comprehensive system that records casemix data** (i.e demographic profiles, patient's encounter on arrival and admissions of patients, diagnosis, treatment, investigations and procedures, discharge numbers, patients' bed days) **from the hospitals to flow into the MOH data pool.**
<u>3) MalaysianDRG Casemix System:</u> Sistem komprehensif yang merekodkan data casemix** (iaitu **data demografik pesakit, data kedatangan dan kemasukan pesakit,** diagnosis, rawatan, penyiasatan dan prosedur, nombor pelepasan, hari tidur pesakit) **dari hospital untuk mengalir ke kumpulan data KKM.

<u>*4)*</u> **<u>Sistem Maklumat Rawatan Pesakit (SMRP):</u> A comprehensive medical treatment report system linking all hospitals in the country.** The required data is entered manually (manual/ some BHIS/IHIS hospitals) or through integration from HIS (most THIS) and will be then integrated into MalaysianDRG Casemix System.
***<u>4) Sistem Maklumat Rawatan Pesakit (SMRP):</u> Sistem laporan rawatan perubatan yang komprehensif menghubungkan semua hospital di negara ini**. Data yang diperlukan dimasukkan secara manual (manual/ beberapa hospital BHIS/IHIS) atau melalui integrasi daripada HIS (kebanyakan hospital THIS) dan kemudiannya akan disepadukan ke dalam Sistem MalaysianDRG Casemix*

Thank you for your cooperation.
Terima kasih atas kerjasama yang telah diberikan.

**SECTIONS**		
**SECTION 1A**<br>**Demographic Profile and Duration of Service**<br>***Profil Demografi dan Pengalaman Bekerja***		Please fill in/tick (√) your response in the appropriate boxes.<br><br>*Sila isikan/tandakan √ pada kotak jawapan pilihan anda.*
**1**	Gender<br>*Jantina*	□ Male (Lelaki)<br>□ Female (Wanita)
**2**	Age (in years)<br>*Umur (dalam tahun)*	____years (tahun)
**3**	Hospital<br>*Hospital*	□Hospital Putrajaya, WP Putrajaya<br>□Hospital Sultanah Nur Zahirah, Kuala Terengganu, Terengganu<br>□Hospital Sultan Ismail, Johor Bahru, Johor<br>□Sultanah Bahiyah, Alor Setar, Kedah<br>□Pusat Jantung Sarawak, Kota Samarahan, Sarawak
**4**	Professional Role or Position<br>*Peranan/Jawatan*	□ Hospital Director (Pengarah Hospital)<br>□ Deputy Director (Timbalan Pengarah)<br>□Consultant Specialist/Specialist (Pakar Perunding/Pakar)<br>□Medical Officer (Pegawai Perubatan)<br>□House Officer (Pegawai Perubatan Siswazah)
**5**	Education Background<br>*Latar Belakang Pendidikan Tertinggi*	□Post-Doctorate (Pasca- Doktor Falsafah)<br>□Philosophy Doctor (PhD)/Ijazah Doktor Falsafah<br>□Sub-Specialty (Sub-Kepakaran)<br>□Master's Degree (Ijazah Sarjana)<br>□Bachelor's Degree (Ijazah Sarjana Muda)
**6**	Duration of years working in Ministry of Health Malaysia (in years)<br><br>*Tempoh perkhidmatan dalam Kementerian Kesihatan Malaysia (dalam tahun)*	____years (tahun)<br><br>____
**7**	Duration of service at current hospital (in years)<br><br>*Tempoh perkhidmatan di hospital ini (dalam tahun)*	_____years (tahun)
**8**	Have you ever undergone training related to the Casemix system during your service at the Ministry of Health?	□ Yes (Ya) □No (Tidak)

	Pernahkah anda menjalani latihan berkaitan sistem Casemix sepanjang perkhidmatan anda di Kementerian Kesihatan?										
	**SECTION 1B**<br>**Knowledge on Casemix (Pengetahuan mengenai Casemix)**	Please indicate your responses based on the scale from 1-10, with 1 being 'no knowledge, and 10 being 'Excellent Knowledge'<br><br>*Sila tandakan jawapan pilihan anda mengikut skala dari 1-10, di mana 1 adalah 'tiada pengetahuan', dan 10 adalah 'pengetahuan yang sangat baik*<br><br>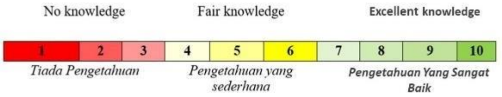									
**1**	Casemix system is one of the MOH's strategy to improve quality of health care in medical and health facilities in Malaysia.<br><br>*Sistem Casemix merupakan salah satu strategi KKM untuk menambahbaik kualiti penjagaan kesihatan difasiliti perubatan dan kesihatan di Malaysia.*	1	2	3	4	5	6	7	8	9	10
**2**	The MalaysianDRG casemix system was introduced by the Ministry of Health (MOH) in October 2010 and subsequently implemented throughout the entire country.<br><br>*Sistem casemix MalaysianDRG telah diperkenalkan oleh Kementerian Kesihatan (KKM) pada Oktober 2010dan seterusnya dilaksanakan di seluruh negara.*	1	2	3	4	5	6	7	8	9	10
**3**	The main objective of Casemix System is meant for estimating the costs which has been spent for each Diagnosis Related Group (DRG) or Main diagnosis Category (MDC).<br><br>*Objektif utama pelaksanaan Sistem Casemix adalah untuk menganggar kos rawatan yang dibelanjakan keatas sesuatu Diagnosis Related Group (DRG) or Main diagnosis Category (MDC).*	1	2	3	4	5	6	7	8	9	10
**4**	Casemix System is involving and evaluating both clinical and costing data.<br><br>*Sistem Casemix melibatkan dan menilai kedua-dua data klinikal dan kewangan.*	1	2	3	4	5	6	7	8	9	10
**5**	Casemix System is involving healthcare workers from various positions and disciplines.<br><br>*Sistem Casemix melibatkan kakitangan kesihatan daripada pelbagai jawatan dan disiplin.*	1	2	3	4	5	6	7	8	9	10
**6**	The implementation of the Casemix system that contains clinical data will involve demographic profiles, patient's encounter on arrivaland admissions of patients, diagnosis, treatment, investigations and procedures.	1	2	3	4	5	6	7	8	9	10

	Pelaksanaan Sistem Casemix yang mengandungi data klinikal akan melibatkan data demografik pesakit,data kedatangan dan kemasukan pesakit, diagnosis,rawatan, ujian-ujian dan prosedur.										
**7**	The implementation of the Casemix system that contains financial data will involve discharge numbers, patients' bed days, encounter/workload for inpatient, percentage of time spent, hospital expenditure, building value, land value, outpatient and daycare, space areas, annual emolument, assets purchased, medical/health/dental supplies, out-of-pockets and medical aid supports.<br><br>*Pelaksanaan sistem Casemix yang mengandungi data kewangan akan melibatkan nombor discaj, hari tidur pesakit, pertemuan/beban kerja untuk pesakitdalam, peratusan masa yang dihabiskan, perbelanjaan hospital, nilai bangunan, nilai tanah, pesakit luar dan jagaan harian, kawasan ruang, emolumen tahunan, aset yang dibeli, bekalan perubatan/kesihatan/pergigian, perbelanjaan luar poket dan sokongan bantuan perubatan.*	1	2	3	4	5	6	7	8	9	10
**8**	The implementation of the Casemix System requires healthcare staff to provide accurate primary diagnosis, complete secondary diagnosis and accurate main procedures, and a complete list of other procedures/ treatment/ investigations.<br><br>*Pelaksanaan Sistem Casemix memerlukan kakitangan penjagaan kesihatan untuk menyediakan diagnosis utama yang tepat, diagnosis lain yanglengkap dan prosedur utama yang tepat, dan senarai lengkap prosedur/rawatan/penyiasatan lain.*	1	2	3	4	5	6	7	8	9	10
**9**	Implementing Casemix System in Total Hospital Information System (THIS) will integrate all required data into the Sistem Maklumat Rawatan Pesakit (SMRP), the MalaysianDRG Casemix System, and finally into Executive Information System.<br><br>*Dengan pelaksanaan Sistem Casemix dalam Sistem Maklumat Hospital Jumlah (THIS), semua data yang diperlukan akan disepadukan ke dalam Sistem MaklumatRawatan Pesakit (SMRP), Sistem Casemix MalaysianDRG dan akhirnya ke dalam Executive Information System.*	1	2	3	4	5	6	7	8	9	10
**10**	Monitoring and evaluation is done through indicators that have been specially prepared for the Casemix System by the Malaysian Ministry of Health through the MalaysianDRG Casemix System application, as well as monitors through the audit function in the system as well as through audit documentation and codes on Per-PD 301.<br><br>*Pemantauan dan penilaian dilakukan melalui indikator-indikator yang telah disediakan khas untuk Sistem Casemix oleh pihak KementerianKesihatanMalaysia melalui aplikasi*	1	2	3	4	5	6	7	8	9	10

	MalaysianDRG Casemix System, serta pemantauan melalui fungsi audit dalam sistem serta melalui audit dokumentasi dan kod pada Per-PD 301										
**SECTION 2**<br>**Critical Success Factors (Faktor-faktor Kejayaan Kritikal)**		Please indicate your responses based on the scale from 1-10, with 1 being 'strongly disagree', *and 10 being 'strongly agree'*<br><br>*Sila tandakan jawapan pilihan anda mengikut skala dari 1-10, di mana 1 adalah 'sangat tidak setuju', dan 10 adalah 'sangat setuju'*<br><br>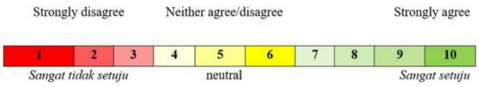									
**PE1**	It is easy to find the information I needed from THIS and/or MalaysianDRG Casemix System.<br><br>*Mudah untuk mencari maklumat yang saya perlukan daripada THIS dan/atau MalaysianDRG Casemix System.*	1	2	3	4	5	6	7	8	9	10
**PE2**	Using Casemix System with THIS is easy in all its steps.<br><br>*Menggunakan dan mengadaptasi Sistem Casemix dengan THIS adalah mudah dalam semua langkahnya.*	1	2	3	4	5	6	7	8	9	10
**PE3**	I find it easy and flexible to get the THIS to do what I want for Casemix purposes.<br><br>*Saya rasa mudah dan fleksibel untuk THIS melakukan apa yang saya kehendaki bagi tujuan Casemix.*	1	2	3	4	5	6	7	8	9	10
**PE4**	It is easy to learn Casemix documentation with THIS support available.<br><br>*Sangat mudah untuk mempelajari penulisan dokumentasi klinikal Casemix dengan sokongan perkhidmatan THIS yang tersedia.*	1	2	3	4	5	6	7	8	9	10
**PE5**	My performance at work has improved as I am using THIS and the Casemix System.<br><br>*Prestasi saya di tempat kerja telah bertambah baik kerana saya menggunakan THIS dan mengadaptasi Sistem Casemix.*	1	2	3	4	5	6	7	8	9	10
**PU1**	I am satisfied with the implementation of the Casemix System in THIS environment.<br><br>*Saya berpuas hati dengan pelaksanaan Sistem Casemix dalam persekitaran THIS.*	1	2	3	4	5	6	7	8	9	10
**PU2**	The use of the Casemix System in THIS environment has facilitated my job operations.<br><br>*Penggunaan Sistem Casemix dalam persekitaran THIS telah memudahkan operasi kerja saya.*	1	2	3	4	5	6	7	8	9	10
**PU3**	Casemix System adoption in THIS context results in more success in achieving job objectives.	1	2	3	4	5	6	7	8	9	10

	Penerimaan Sistem Casemix dalam konteks THIS menghasilkan lebih banyak kejayaan dalam mencapai objektif pekerjaan.										
**PU4**	I find the integration of THIS and/or SMRP and/or MalaysianDRG Casemix System useful in my job.<br><br>*Saya mendapati integrasi THIS dan/atau SMRP dan/atau MalaysianDRG Casemix System berguna dalam tugas saya.*	1	2	3	4	5	6	7	8	9	10
**O1**	Organizational competency to provide the resources for the implementation of the Casemix System is required in THIS setting.<br><br>*Kecekapan organisasi untuk menyediakan sumber untuk pelaksanaan Sistem Casemix diperlukan dalam tetapan THIS*	1	2	3	4	5	6	7	8	9	10
**O2**	My employer and my head of department is supportive to me for my work involving THIS and Casemix.<br><br>*Majikan dan ketua jabatan saya menyokong saya menyokong saya untuk kerja saya yang melibatkan THIS dan Casemix.*	1	2	3	4	5	6	7	8	9	10
**O3**	Casemix System adoption in THIS settings is supported by Ministry of Health, State Health Department and the hospital top management.<br><br>*Pelaksanaan Sistem Casemix dalam tetapan THIS disokong oleh Kementerian Kesihatan Malaysia, Jabatan Kesihatan Negeri dan pengurusan tertinggi hospital.*	1	2	3	4	5	6	7	8	9	10
**O4**	Hospital top management is responsible to provide training for Casemix and THIS.<br><br>*Pengurusan tertinggi hospital bertanggungjawab menyediakan latihan untuk Sistem Casemix dan THIS.*	1	2	3	4	5	6	7	8	9	10
**O5**	Series of training on the clinical documentation and costing module according to the stipulated guidelines prepared by the Ministry of Health Malaysia are adequate.<br><br>*Siri latihan dokumentasi klinikal dan modul kewangan mengikut garis panduan yang ditetapkan yang disediakan oleh Kementerian Kesihatan Malaysia adalah mencukupi.*	1	2	3	4	5	6	7	8	9	10
**O6**	Organizational competency leads to the easiness of Casemix System adoption in THIS context.<br><br>*Kecekapan organisasi membawa kepada kemudahan penggunaan Sistem Casemix dalam konteks THIS.*	1	2	3	4	5	6	7	8	9	10
**O7**	Organizational competency leads to the usefulness of Casemix System adoption in THIS context.<br><br>*Kecekapan organisasi membawa kepada kegunaan penggunaan Sistem Casemix dalam konteks THIS.*	1	2	3	4	5	6	7	8	9	10
**O8**	The service providers of THIS and/or MalaysianDRG Casemix System adequately provide training to the users.	1	2	3	4	5	6	7	8	9	10

	Pembekal perkhidmatan THIS dan/atau MalaysianDRG Casemix System menyediakan latihan secukupnya kepada pengguna.										
**O9**	Adequate technical/application support is provided for Casemix System implementation in THIS context.<br><br>*Sokongan teknikal/aplikasi yang mencukupi disediakan untuk pelaksanaan Sistem Casemix dalam konteks THIS.*	1	2	3	4	5	6	7	8	9	10
**SQ1**	The THIS and/or MalaysianDRG Casemix System are always available.<br><br>*THIS dan/atau MalaysianDRG Casemix System sentiasa tersedia.*	1	2	3	4	5	6	7	8	9	10
**SQ2**	The THIS and/or MalaysianDRG Casemix System are user-friendly.<br><br>*THIS dan/atau MalaysianDRG Casemix System adalah mesra pengguna.*	1	2	3	4	5	6	7	8	9	10
**SQ3**	The THIS and/or MalaysianDRG Casemix System provide interaction between users and the system.<br><br>*THIS dan/atau MalaysianDRG Casemix System menyediakan interaksi antara pengguna dan sistem.*	1	2	3	4	5	6	7	8	9	10
**SQ4**	The THIS and/or MalaysianDRG Casemix System provide high-speed information access.<br><br>*THIS dan/atau MalaysianDRG Casemix System menyediakan akses maklumat berkelajuan tinggi.*	1	2	3	4	5	6	7	8	9	10
**IQ1**	The information required for the Casemix system generated by THIS is accurate and correct.<br><br>*Maklumat yang diperlukan untuk Sistem Casemix dijana oleh THIS adalah tepat dan betul.*	1	2	3	4	5	6	7	8	9	10
**IQ2**	The information generated by the THIS is useful for Casemix purposes by providing complete information.<br><br>*Maklumat yang dijana oleh THIS berguna untuk tujuan Casemix dengan menyediakan maklumat lengkap*	1	2	3	4	5	6	7	8	9	10
**IQ3**	The THIS generates information for Casemix purposes in a timely manner.<br><br>*THIS menjana maklumat untuk tujuan Casemix tepat pada masanya.*	1	2	3	4	5	6	7	8	9	10
**IQ4**	The information needed for Casemix is available all the time with THIS.<br><br>*Maklumat yang diperlukan untuk Casemix tersedia sepanjang masa dengan THIS.*	1	2	3	4	5	6	7	8	9	10
**IQ5**	I trust the information output of the THIS and/or MalaysianDRG Casemix System.<br><br>*Saya mempercayai output maklumat bagi Sistem THIS dan/atau Casemix.*	1	2	3	4	5	6	7	8	9	10

**SE1**	The THIS can be relied on to provide information as and when needed for Casemix purposes.<br><br>*THIS boleh dipercayai untuk memberikan maklumat apabila diperlukan untuk tujuan Casemix.*	1	2	3	4	5	6	7	8	9	10
**SE2**	The vendor support services of the THIS and/or SMRP and/or MalaysianDRG Casemix System provide sufficient technical assistance.<br><br>*Penyedia Sistem Casemix THIS dan/atau SMRP dan/atau MalaysianDRG menyediakan bantuan teknikal yang mencukupi.*	1	2	3	4	5	6	7	8	9	10
**SE3**	If those vendor support services promise to do something by a specific time, they will.<br><br>*Jika perkhidmatan sokongan berjanji untuk melakukan sesuatu pada masa tertentu, mereka akan melakukannya.*	1	2	3	4	5	6	7	8	9	10
**SE4**	The output from the integration of THIS and/or SMRP and/or MalaysianDRG Casemix system completes the Casemix workflow.<br><br>*Output daripada integrasi THIS dan/atau SMRP dan/atau MalaysianDRG Casemix System* melengkapkan proses kerja Casemix.	1	2	3	4	5	6	7	8	9	10
**SE5**	The overall infrastructure in place is adequate to support THIS services and the Casemix System.<br><br>*Infrastruktur keseluruhan yang disediakan adalah mencukupi untuk menyokong THIS dan Sistem Casemix.*	1	2	3	4	5	6	7	8	9	10
**SECTION 3**<br>**(Intention to Use and User Acceptance)**		Please indicate your responses based on the scale from 1-10, with 1 being 'strongly disagree', *and 10 being 'strongly agree'*<br><br>*Sila tandakan jawapan pilihan anda mengikut skala dari 1-10, di mana 1 adalah 'sangat tidak setuju', dan 10 adalah 'sangat setuju'*<br><br>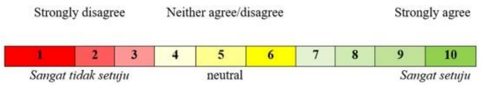 									
**ITU1**	I enjoy and motivated to work in a THIS hospital where the Casemix System is being implemented.<br><br>*Saya suka dan bermotivasi untuk bekerja di hospital yang dilengkapi dengan THIS di mana Sistem Casemix sedang dilaksanakan.*	1	2	3	4	5	6	7	8	9	10
**ITU2**	I think the implementation of the Casemix System with THIS-provided services is a good idea.<br><br>*Saya merasakan pelaksanaan Sistem Casemix dengan perkhidmatan yang disediakan THIS adalah idea yang baik.*	1	2	3	4	5	6	7	8	9	10
**ITU3**	I believe the hospital staff is receptive to the implementation of the Casemix system.	1	2	3	4	5	6	7	8	9	10

	Saya percaya kakitangan hospital menerima pelaksanaan Sistem Casemix.										
**ITU4**	I agree with the idea of implementing Casemix System and extending it to other THIS hospital.<br><br>*Saya bersetuju dengan idea untuk melaksanakan Sistem Casemix dan memanjangkannya ke hospital yang dilengkapi dengan THIS yang lain*	1	2	3	4	5	6	7	8	9	10
**ITU5**	I recommend using THIS to support Casemix System implementation.<br><br>*Saya mengesyorkan penggunaan THIS bagi menyokong pelaksanaan Sistem Casemix.*	1	2	3	4	5	6	7	8	9	10
**A1**	Casemix System implementation with THIS setting facilitates easy access to patient information.<br><br>*Pelaksanaan Sistem Casemix dengan tetapan THIS memudahkan akses mudah kepada maklumat pesakit.*	1	2	3	4	5	6	7	8	9	10
**A2**	Casemix System adoption with THIS provided service enables me to accomplish tasks more efficiently and increase my quality of work.<br><br>*Pelaksanaan Sistem Casemix dalam tetapan THIS membolehkan saya menyelesaikan tugas dengan lebih cekap dan meningkatkan kualiti kerja saya.*	1	2	3	4	5	6	7	8	9	10
**A3**	The implementation of the Casemix system in THIS setting contributes to more accurate and complete diagnosis and procedures.<br><br>Pelaksanaan Sistem Casemix dalam tetapan THIS menyumbang kepada diagnosis dan prosedur yang lebih tepat dan lengkap.	1	2	3	4	5	6	7	8	9	10
**A4**	The integration of Casemix System and THIS will help overcome the limitations of the paper-based system.<br><br>*Penyepaduan/Integrasi MalaysianDRG Casemix System dan/atau THIS akan membantu mengatasi batasan sistem berasaskan kertas.*	1	2	3	4	5	6	7	8	9	10
**A5**	Overall, I am satisfied with the Casemix System implementation in THIS setting.<br><br>*Secara keseluruhannya, saya berpuas hati dengan pelaksanaan Sistem Casemix dalam tetapan THIS.*	1	2	3	4	5	6	7	8	9	10
