# Supplementary material for: Critical Success Factors and Acceptance of the Casemix System Implementation Within the Total Hospital Information System: Exploratory Factor Analysis of a Pilot Study
Source: JMIR Form Res. 2024 Oct 29;8:e56898. doi: 10.2196/56898 (PMC11558226; doi:10.2196/56898)
Supplement: Multimedia Appendix 7 [file formative_v8i1e56898_app7.pdf]

INFORMED CONSENT FORM
(QUESTIONNAIRE)

Title of Study: Critical Success Factors and The Acceptance of Casemix System in the Total Hospital Information System of the Ministry of Health Malaysia

By signing below, I confirm the following:

- I have been given oral and written information for the above study and have read and understood the information given.
- I have had sufficient time to consider participation in the study and have had the opportunity to ask questions and all my questions have been answered satisfactorily.
- I understand that my participation is voluntary and I can at any time free withdraw from the study without giving a reason and this will in no way affect my future treatment. I am not taking part in any other research study at this time. I understand the risks and benefits, and I freely give my informed consent to participate under the conditions stated.
- I understand that if I decide to withdraw from the study midway, I could exit the site freely and no measures will be used to preserve the data I have filled in, thus all data will be destroyed.
- I understand that I must follow the study investigator’s instructions related to my participation in the study.
- I understand that study staff, qualified monitors and auditors, the sponsor or its affiliates, and governmental or regulatory authorities, have direct access to my medical record in order to make sure that the study is conducted correctly and the data are recorded correctly. All personal details will be treated as **STRICTLY CONFIDENTIAL.**
- I consent to provide my personal details and that the information will be managed ethically according to the terms of the Malaysian Personal Data Protection Act 2010 (PDPA).
- I will receive a copy of this subject information/informed consent form via e-mail.
- I understand that anonymity and confidentiality will be maintained throughout the duration of the study. It is not possible to identify me in any publication.
- I understand that the outcome of the study may be published in a journal and/or as an internal report for stakeholders. I can have access to these publications or reports.
- I understand that the data will be kept by the researcher securely for the duration of the study and deleted by 31st May 2028.
- I agree/disagree* to participate in this study. *(*delete which is not applicable)*

Subject:

Signature: I/C number:

Name: Date:

Investigator conducting informed consent:

Signature: I/C number:

Name: Date:

If you have any questions about the study or want information about this study, please contact:

Principal Investigator
Dr. Noor Khairiyah binti Mustafa
Jabatan Perubatan Kesihatan Awam
Universiti Kebangsaan Malaysia
Tel: 012-6161342
Email: P115190@siswa.ukm.edu.my

For enquiries regarding the rights of the subject

Jawatankuasa Etika & Penyelidikan Perubatan
(Medical Research & Ethics Committee)
Kementerian Kesihatan Malaysia
Kompleks Institut Kesihatan Negara (NIH),
No.1, Jalan Setia Murni U13/52,
Seksyen U13 Bandar Setia Alam,
40170 Shah Alam, Selangor.
No.Tel: 03-33628407/33628205/ 33628888

Jawatankuasa Etika Penyelidikan Perubatan UKM
Sekretariat Etika Penyelidikan Universiti Kebangsaan Malaysia,
Tingkat 1, Blok Klinikal,
Hospital Canselor Tuanku Muhriz,
Pusat Perubatan UKM,
Jalan Yaacob Latif,
Bandar Tun Razak,
56000 Cheras Kuala Lumpur.
Tel: +603-9145 5046 / 9145 5048
E-mail: sepukm@ukm.edu.my

BORANG PERSETUJUAN/ KEIZINAN PESERTA
(BORANG KAJI SELIDIK)

Tajuk Penyelidikan : *Critical Success Factors and The Acceptance of Casemix System Implementation in Total Hospital Information System of The Ministry of Health Malaysia* (Faktor Kejayaan Kritikal Dan Penerimaan Bagi Pelaksanaan Sistem Casemix di Sistem Maklumat Hospital Bersepadu Di Kementerian Kesihatan Malaysia)

Dengan menandatangani di bawah, saya mengesahkan bahawa:

- Saya telah diberi maklumat tentang penyelidikan di atas secara lisan dan bertulis and saya telah membaca & memahami segala maklumat yang diberikan dalam risalah ini.
- Saya telah diberikan masa yang secukupnya untuk mempertimbangkan penyertaan saya dalam penyelidikan ini dan telah diberi peluang untuk bertanyakan soalan dan semua persoalan saya telah dijawab dengan sempurna dan memuaskan.
- Saya juga faham bahawa penyertaan saya adalah secara sukarela dan pada bila-bila masa saya bebas menarik diri daripada penyelidikan ini tanpa harus memberi sebarang alasan dan ianya sama sekali tidak akan menjejaskan perkhidmatan saya
- Saya tidak mengambil bahagian dalam mana-mana penyelidikan lain pada masa ini. Saya juga memahami tentang risiko dan manfaat penyelidikan ini dan saya secara sukarela memberi persetujuan untuk menyertai penyelidikan ini di bawah syarat-syarat yang telah dinyatakan di atas.
- Saya faham bahawa jika saya memutuskan untuk menarik diri daripada kajian di tengah-tengah, saya boleh keluar dari lokasi temuramah dengan bebas dan tiada langkah akan digunakan untuk mengekalkan data yang telah saya isi, justeru semua data akan dimusnahkan.
- Saya faham saya harus mematuhi nasihat dan arahan yang berkaitan dengan penyertaan saya dalam penyelidikan ini daripada doktor penyelidikan (penyelidik).
- Saya faham bahawa kakitangan penyelidikan, pemantau dan juruaudit terlatih, dan pihak berkuasa kerajaan atau undang-undang, mempunyai akses langsung dan boleh menyemak data peribadi saya bagi memastikan penyelidikan ini dijalankan dengan betul dan data direkodkan dengan betul. Segala maklumat dan data peribadi akan dianggap sebagai **SULIT.**
- Saya bersetuju untuk memberikan butiran peribadi saya dan maklumat tersebut akan diuruskan secara beretika mengikut terma Akta Perlindungan Data Peribadi Malaysia 2010 (PDPA).)
- Saya akan menerima satu salinan ‘Risalah Maklumat Pesakit dan Borang Persetujuan atau Keizinan Pesakit’ yang telah lengkap melalui e-mel.
- Saya faham bahawa kerahsiaan dan kerahsiaan akan dikekalkan sepanjang tempoh kajian. Tidak mungkin untuk mengenal pasti saya dalam mana-mana penerbitan.)
- Saya faham bahawa hasil kajian mungkin diterbitkan dalam jurnal dan/atau sebagai laporan dalaman untuk pihak berkepentingan. Saya boleh mempunyai akses kepada penerbitan atau laporan ini.)
- Saya faham bahawa data akan disimpan oleh penyelidik dengan selamat sepanjang tempoh kajian dan dipadamkan selewat-lewatnya pada 31 Mei 2028.)
- Saya bersetuju/ tidak bersetuju* untuk menyertai penyelidikan ini. (*Potong mana yang tidak berkenaan)

<u>Subjek</u> :

Tandatangan:	Nombor K/P:
Nama:	Tarikh :

<u>Penyelidik yang mengendalikan proses menandatangani borang keizinan:</u>

Tandatangan:	Nombor K/P:
Nama:	Tarikh :

Anda boleh menghubungi Penyelidik Utama kajian ini sekiranya anda mempunyai sebarang pertanyaan mengenai penyelidikan ini:

Penyelidik Utama

Dr. Noor Khairiyah binti Mustafa
Jabatan Perubatan Kesihatan Awam
Universiti Kebangsaan Malaysia
Tel: 012-6161342
Email: P115190@siswa.ukm.edu.my

Jika anda mempunyai sebarang pertanyaan berkaitan dengan hak-hak anda sebagai peserta dalam penyelidikan ini, sila hubungi:

Jawatankuasa Etika & Penyelidikan Perubatan
(Medical Research & Ethics Committee)
Kementerian Kesihatan Malaysia
Kompleks Institut Kesihatan Negara (NIH),
No.1, Jalan Setia Murni U13/52,
Seksyen U13 Bandar Setia Alam,
40170 Shah Alam, Selangor.
No.Tel: 03-33628407/33628205/ 33628888

Jawatankuasa Etika Penyelidikan Perubatan UKM
Sekretariat Etika Penyelidikan Universiti Kebangsaan Malaysia,
Tingkat 1, Blok Klinikal,
Hospital Canselor Tuanku Muhriz,
Pusat Perubatan UKM,
Jalan Yaacob Latif,
Bandar Tun Razak,
56000 Cheras Kuala Lumpur.
Tel: +603-9145 5046 / 9145 5048
E-mel: sepukm@ukm.edu.my
