# Supplementary material for: Critical Success Factors and Acceptance of the Casemix System Implementation Within the Total Hospital Information System: Exploratory Factor Analysis of a Pilot Study
Source: JMIR Form Res. 2024 Oct 29;8:e56898. doi: 10.2196/56898 (PMC11558226; doi:10.2196/56898)
Supplement: Multimedia Appendix 9 [file formative_v8i1e56898_app9.pdf]

Multimedia Appendix 9: The number of items for each construct before and after EFA

No. of construct	Name of Construct	Number of Items before EFA	Number of Items Dropped	Number of Items Retained After EFA
1	System Quality	4	-	4
2	Information Quality	5	-	5
3	Service Quality	5	-	5
4	Organizational Factors	9	1	8
5	Perceived Ease of Use	5	-	5
6	Perceived Usefulness	4	-	4
7	Intention to Use	5	-	5
8	Acceptance	5	-	5
	**Total**	**42**	**1**	**41**
